# Extracellular Vesicles Coordinate Bacterial Cloaking in Lung Epithelial Cells to Alleviate Acute Inflammatory Injury

**DOI:** 10.1002/jev2.70238

**Published:** 2026-02-08

**Authors:** Feng Ding, Shengkai Gong, Haotian Luo, Dandan Wu, Xiaoshan Yang, Zihan Li, Dingmei Zhang, Peijie He, Jiani Liu, Lili Bao, Yang Zhou, Zhengyan Wang, Siying Liu, Pei Wang, Geng Dou, Shiyu Liu

**Affiliations:** ^1^ State Key Laboratory of Oral & Maxillofacial Reconstruction and Regeneration, National Clinical Research Center for Oral Diseases, Shaanxi Key Laboratory of Stomatology, Department of Oral Biology & Clinic of Oral Rare Diseases and Genetic Disease, School of Stomatology The Fourth Military Medical University Xi'an Shaanxi China; ^2^ State Key Laboratory of Oral & Maxillofacial Reconstruction and Regeneration, National Clinical Research Center for Oral Diseases, Shaanxi Key Laboratory of Stomatology, Department of Radiology, School of Stomatology The Fourth Military Medical University Xi'an Shaanxi China; ^3^ State Key Laboratory of Oral & Maxillofacial Reconstruction and Regeneration, National Clinical Research Center for Oral Diseases, Shaanxi International Joint Research Center for Oral Diseases, Center for Tissue Engineering, School of Stomatology The Fourth Military Medical University Xi'an Shaanxi China; ^4^ Department of Orthopaedic Surgery Affiliated Hospital of Zunyi Medical University Zunyi Guizhou China; ^5^ State Key Laboratory of Oral & Maxillofacial Reconstruction and Regeneration, National Clinical Research Center for Oral Diseases, Shaanxi Key Laboratory of Stomatology, Department of Periodontology School of Stomatology The Fourth Military Medical University Xi'an Shaanxi China; ^6^ State Key Laboratory of Oral & Maxillofacial Reconstruction and Regeneration, National Clinical Research Center for Oral Diseases, Shaanxi Key Laboratory of Stomatology, Digital Center, School of Stomatology The Fourth Military Medical University Xi'an Shaanxi China; ^7^ State Key Laboratory of Oral & Maxillofacial Reconstruction and Regeneration, National Clinical Research Center for Oral Diseases, Shaanxi Key Laboratory of Stomatology Department of Orthodontics, School of Stomatology The Fourth Military Medical University Xi'an Shaanxi China

**Keywords:** acute bacterial infection, extracellular vesicles, EV‐bacteria complexes, pathogen cloaking, *Staphylococcus aureus*

## Abstract

The capacity of host professional phagocytes to attenuate excessive inflammatory responses through pathogen cloaking during infection has been well‐established. However, the involvement of non‐professional phagocytes in this process remains unknown. Here, we identify a previously unrecognized mechanism by which lung epithelial cells (LECs) attenuate inflammatory responses during *Staphylococcus aureus* infection. *S. aureus*‐challenged LECs rapidly shed extracellular vesicles (EVs) carrying surface receptors capable of binding invading bacteria and forming EV‐bacteria complexes. The EV‐bacteria complexes were internalized by LECs via RhoA‐ROCK1‐actin‐driven endocytosis pathway, reducing free bacterial burden within the alveolar lumen. This EV‐mediated pathogen cloaking conferred acute‐phase protection, as demonstrated by mitigating early‐stage pulmonary inflammation, and improving survival rates in infected mice. Paradoxically, this strategy permitted chronic bacterial persistence and sustaining low‐grade inflammation. Our findings delineate a trade‐off mechanism that non‐professional phagocytes modulate acute bacterial infection and inflammatory responses via pathogen cloaking. This mechanistic perspective reframes non‐professional phagocytes as active architects of infection outcomes based on EV‐mediated host‐pathogen interactions. Our work provides insights into the mechanism of bacterial cloaking during infection and suggests stage‐specific therapeutic strategies.

## Graphical abstract

Schematic diagram of lung epithelial cell‐derived extracellular vesicles mediating bacterial cloaking to attenuate acute inflammatory injury. Lung epithelial cells attenuate early *S. aureus* infection by shedding extracellular vesicles that capture bacteria via surface receptors. These EV‐bacteria complexes are internalized through a RhoA‐ROCK1‐actin pathway, reducing acute inflammation but enabling chronic persistence. This study delineates a trade‐off cloaking mechanism for non‐professional phagocytes in shaping infection outcomes.

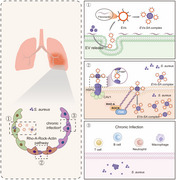

## 1 Introduction

The pulmonary immune system dynamically balances pathogen clearance with inflammation regulation to maintain tissue homeostasis during infection. Epithelial alarmins, resident alveolar macrophages (AMs), and neutrophil recruitment constitute the canonical defense cascade for eliminating bacteria during infection (Robb et al. 2016). However, excessive neutrophilic infiltration triggered by the overwhelming bacterial burden often culminates in acute lung injury (ALI), a leading cause of mortality in severe pulmonary infections (Grommes and Soehnlein 2011). Emerging evidence reveals that AMs actively prevent neutrophil hyperactivation to curb uncontrolled inflammatory cascades by phagocytosing and cloaking *S. aureus* (Neupane et al. 2020). In addition, pleural macrophages have also been demonstrated to execute similar inflammation regulation functions and improve outcomes by translocating to infected lung tissue (Stumpff et al. 2023). These findings highlight a unique immunomodulatory pathway operating independently of classical immune activation to alleviate pathological lung injury. Despite the crucial role of macrophages in the regulation of pulmonary infection and inflammation, the significant numerical disparity, with only one macrophage present in every three alveoli, critically limits their effectiveness in resisting bacteria invasion during acute infection (Neupane et al. 2020). Therefore, the definite cellular mechanisms governing early‐phase pathogen cloaking and inflammation regulation remain incompletely understood.

The extensive infiltration of bacterial pathogens into the alveolar space during pulmonary infection exposes lung epithelial cells (LECs) to direct microbial challenge (Hook et al. 2018). As the sentinel interface of pulmonary immunity, LECs confront invading pathogens through multifaceted defensive strategies. It is now well‐established that LECs orchestrate an integrated network of defense mechanisms, including the physical mucus barrier, antimicrobial peptide secretion and synchronized immune signalling cascades (Whitsett and Alenghat 2015). This concerted response effectively restricts bacterial invasion, colonization and uncontrolled inflammatory amplification. Notably, while recent observations have unveiled phagocytosis of bacteria by epithelial cells (Prystopiuk et al. 2018), whether this process serves a pathogen cloaking function analogous to professional phagocytes remains undefined. Deciphering the spatiotemporal dynamics and molecular determinants of these epithelial‐pathogen crosstalk holds promise to revolutionize therapeutic strategies for managing infectious diseases.

Current trends indicate that organism‐derived vesicle‐like structures mediate dynamic interactions between host and pathogen via context‐dependent molecular pathways (Berry et al. 2022; Tey et al. 2022). Host‐derived extracellular vesicles (hEVs) often undergo stimulus‐responsive defensive modifications upon pathogen exposure (Keller et al. 2020). Based on the principle of surface‐area‐to‐volume ratio, EVs exhibit significantly stronger efficacy than parental cells through surface structures or receptors, enabling amplified molecular interactions with pathogens (Carney et al. 2025). Existing evidence shows that hEVs play dual roles during bacterial infection, both mediating protective immunity and enabling pathogen dissemination and invasion (Zou et al. 2022). On the one hand, hEVs combat bacterial threats through virulence factor neutralization (Keller et al. 2020), nutrient sequestration (Kuang et al. 2023) and immunogenic antigen presentation (Ramachandra et al. 2010). On the other hand, hEVs were exploited by pathogens as vehicles for horizontal transfer of virulence factors (Abrami et al. 2013; Nandakumar et al. 2019). Even tumours are capable of domesticating microorganisms using EVs of their own origin to facilitate tumour progression (Jiang et al. 2022). Despite substantial progress in deciphering EV‐mediated host‐pathogen crosstalk (Arteaga‐Blanco and Bou‐Habib 2021; Erttmann et al. 2022), the paradoxical function of hEVs in facilitating bacterial internalization while simultaneously supporting host defense remains ambiguous.

Here, we unveil an immunoregulatory axis in which LECs coordinate bacterial cloaking to prevent acute lethal pulmonary inflammation during *S. aureus* infection by their released EVs. In the acute pneumonia mice model induced by *S. aureus*, *S. aureus* stimulates the release of EVs from LECs that carry surface receptors to bind free *S. aureus*. The resulting *EVs‐S. aureus* complex (*EVs‐SA*) is internalized by host LECs via RhoA‐ROCK1‐actin‐driven endocytosis pathway, promoting the cloaking of bacteria within the LECs. This strategic pathogen cloaking significantly reduces pulmonary inflammation and improves survival outcomes in lethally infected mice, but at the cost of the persistent inflammation caused by long‐term bacteria colonization and chronic infections (Graphical Abstract). Collectively, these findings delineate a previously unrecognized regulatory pathway through which epithelial barriers dynamically modulate infection outcomes through EVs‐mediated pathogen cloaking. This EVs‐mediated bacteria cloaking strategy represents an evolutionary trade‐off that transiently compromising pathogen clearance to prevent fatal immunopathology. This study provides insights into the mechanisms of bacterial cloaking during infection and suggests stage‐specific therapeutic strategies.

## 2 Results

### 2.1 Inhibition of LuEV Release Reduces Intracellular Bacterial Colonization and Aggravates Lung Inflammation in *S. aureus*‐Induced Pneumonia

**FIGURE 1:**
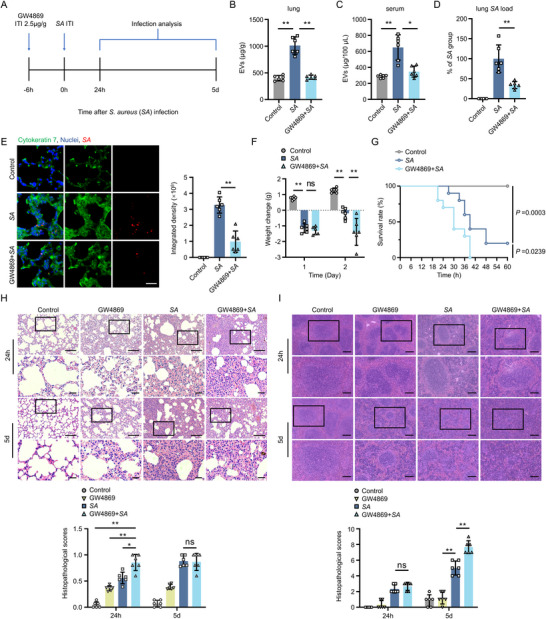
Inhibition of LuEV release by GW4869 reduces bacterial colonization and exacerbates pulmonary inflammatory injury. (A) Schematic of the experimental design for detecting infection levels in *S. aureus*‐infected mice with EV release inhibited by GW4869. (B, C) The EV level in lung tissue (B) and serum (C) of infected mice in different groups at 24 h post‐*S. aureus* infection (*n* = 6). (D) Quantification of lung tissue‐resident bacteria at 24 h post‐*S. aureus* infection (*n* = 6). (E) Representative fluorescence images and quantitative analysis of *S. aureus* (red) level in cytokeratin 7^+^ LECs (green) in lung tissue of infected mice from different groups at 24 h post‐*S. aureus* infection (*n* = 6). Scale bar, 25 µm. (F) Body weight monitoring in sublethally infected mice (1 × 10^8^ CFU *S. aureus*) treated with or without GW4869 (*n* = 6). (G) Survival analysis of lethally infected mice (6 × 10^8^ CFU *S. aureus*) treated with or without GW4869 (*n* = 10). (H, I) Hematoxylin and eosin (H&E) staining and histopathological score of lungs (H) and spleen tissue (I) in infected mice from different groups at 24 h or 5 days post‐infection (*n* = 6). Scale bar, 100 µm (low‐magnification) and 25 µm (high‐magnification) (H); 200 µm (low‐magnification) and 100 µm (high‐magnification) (I). ITI denotes intratracheal instillation. *SA* denotes *Staphylococcus aureus*. Data are presented as mean ± SD. One‐way ANOVA with Tukey's post hoc test was used for statistical analysis of (B–F, H, I), and the log‐rank test for (G). *P < 0.05, **P < 0.01.

Host‐derived extracellular vesicles (hEVs) represent a critical responsive mechanism of host cells during infection, orchestrating complex immunomodulatory processes to influence disease progression (Zou et al. 2022). In the well‐established acute pneumonia mice model induced by *S. aureus* (1 × 10^8^ CFU) via intratracheal instillation (Figure 1A), we observed a significant increase in EV levels in both lung tissues and circulation (Figure 1B,C), consistent with previous reports linking infection to EV upregulation. To investigate the role of lung tissue‐derived extracellular vesicles (LuEVs) in pulmonary infection, pharmacological inhibition of EV release was achieved through administration of GW4869, a well‐characterized inhibitor of EV biogenesis with validated efficacy in both in vitro and in vivo models (Iguchi et al. 2016; Kuang et al. 2023). A single intrapulmonary instillation of GW4869 at 6 h prior to infection was found to effectively reduce EV levels in both lung tissues and circulation within 24 h post‐infection (h.p.i.) (Figure 1B,C). This reduction in EVs coincided with a marked decrease in tissue‐resident *S. aureus* colonization level (Figure 1D). Immunofluorescence analysis of lung tissues further confirmed that inhibition of LuEV release reduced the *S. aureus* internalized by LECs (Figure 1E), which was determined following lysostaphin treatment to eliminate extracellular free bacteria (Figure S1). These findings appear to indicate that LuEVs may facilitate bacterial internalization and intracellular colonization. To systematically assess the functional impact of LuEVs during pulmonary infection, disease progression was longitudinally monitored through quantitative measurement of body weight and survival analysis in infected mice receiving different therapeutic regimens. Mice infected with sublethal doses of *S. aureus* demonstrated significant body weight loss at 24 h.p.i., with a gradual recovery of weight observed at 48 h.p.i. (Figure 1F). Meanwhile, mice infected with lethal doses exhibited 80% mortality at 60 h.p.i. (Figure 1G). Pretreatment with GW4869 delayed weight recovery of the infected mice after sublethal infection (Figure 1F), and they experienced 100% mortality by 40 h post‐lethal challenge (Figure 1G). Notably, while inhibiting EV release during infection effectively reduced intracellular bacterial burdens in LECs, *S. aureus*‐infected mice models exhibited a paradoxical increase in mortality rates. This unanticipated inverse relationship between intracellular bacterial colonization and host survival necessitates deeper exploration of the underlying pathophysiological mechanisms.

Next, systematic analysis of pulmonary inflammation and tissue damage was conducted in *S. aureus*‐infected mice following inhibition of LuEV release. Histological analysis revealed that GW4869 pretreatment exacerbated *S. aureus*‐induced lung damage during the acute phase (24 h.p.i.), with near‐complete alveolar destruction, haemorrhagic consolidation and inflammation infiltration (Figure 1H). Statistical analysis demonstrated that GW4869‐treated infected cohorts displayed a significant increase in histopathological scores at 24 h.p.i. compared with *S. aureus*‐infected mice, whereas no significance was observed by 5 days post‐infection (d.p.i.), indicating acute phase‐specific potentiation of pulmonary inflammation mediated by the inhibition of LuEV release (Figure 1H). Immunofluorescence also confirmed elevated neutrophil recruitment, macrophage infiltration and C‐reactive protein (CRP) expression in the lungs of GW4869‐treated *S. aureus*‐infected mice at both 24 h.p.i. and 5 d.p.i., underscoring GW4869 pretreatment exacerbates the pulmonary inflammatory response during *S. aureus* infection (Figure S2A–C). Moreover, it was noted that pulmonary bacterial infection caused the upregulation of circulating EVs level, and the systemic inflammatory damage was also examined. Although no significant difference in histopathological scores of splenic lesions was observed between GW4869‐treated and untreated *S. aureus*‐infected groups at 24 h.p.i., GW4869 also led to marked architectural disruption and inflammatory foci formation in splenic parenchyma by 5 d.p.i. (Figure 1I). These results demonstrated that pharmacological inhibition of host‐derived LuEV release significantly exacerbated inflammatory responses in lung during *S. aureus* infection, thereby elevating infection‐associated mortality.

Altogether, these findings indicate that inhibition of LuEV release reduced *S. aureus* colonization within LECs and exacerbated early‐stage inflammation in lung, providing direct evidence that EVs derived from lung tissue mediate *S. aureus* internalization by host LECs during the early stages of infection. However, the role and underlying mechanisms of EVs in this process require further investigation.

### 2.2 Lung Epithelial Cell‐EVs Mediate Intracellular *S. aureus* Cloaking and Attenuation of Inflammatory Damage

**FIGURE 2:**
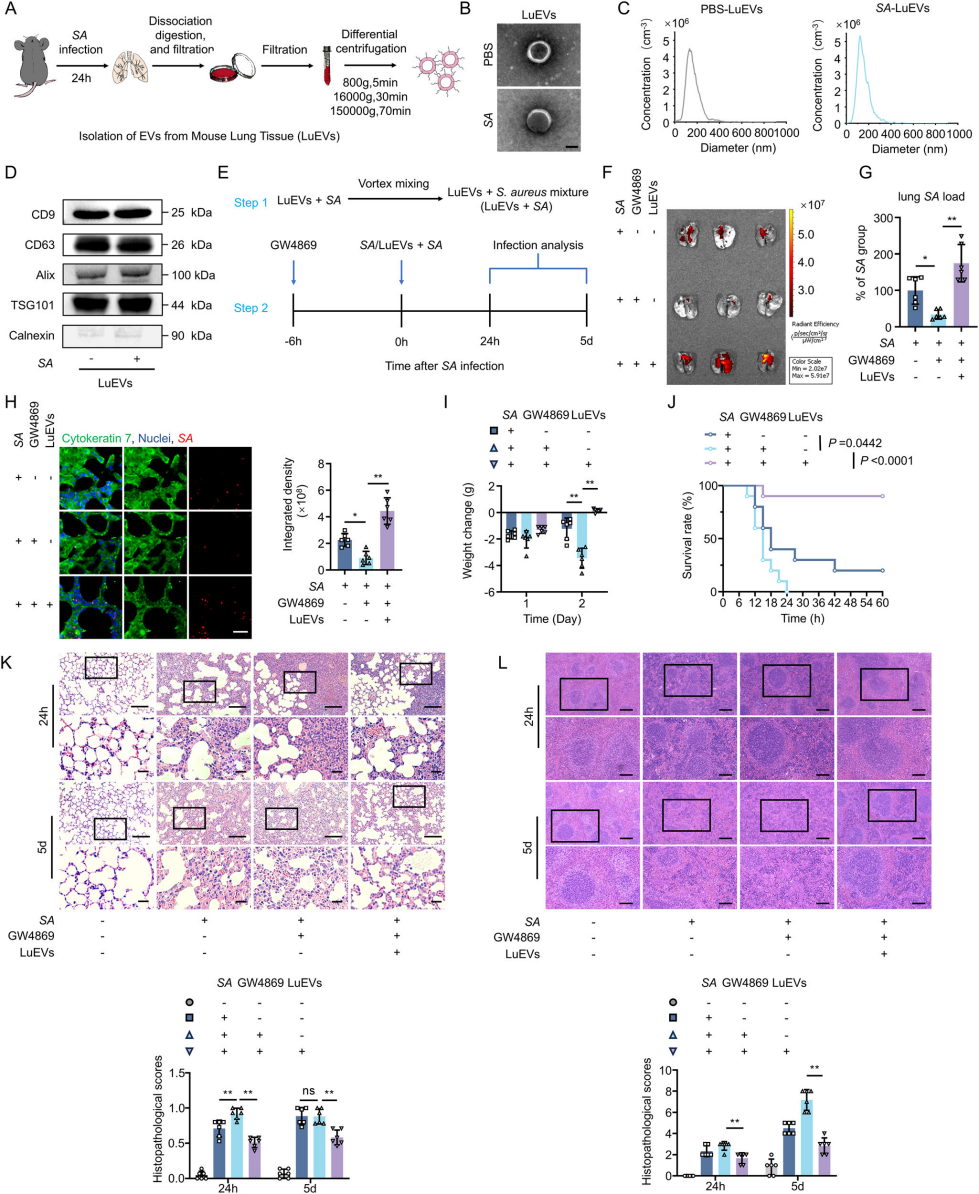
LuEVs promote *S. aureus* colonization while reducing pulmonary inflammation and mortality. (A) Schematic diagram of the isolation procedure for EVs from mouse lung tissue (LuEVs). (B) TEM images of LuEVs from uninfected mice (PBS‐LuEVs) and *S. aureus*‐infected mice (*SA*‐LuEVs). Scale bar, 200 nm. (C) Size distribution curve of PBS‐LuEVs and *SA*‐LuEVs by NTA. (D) Western blot analysis of EVs markers. (E) Schematic diagram of LuEVs + *SA* mixture (LuEVs + *SA*) preparation and in vivo application in mouse infection studies. (F) Representative biodistribution of mCherry‐labelled *S. aureus* in mouse lung tissue at 24 h.p.i. (*n* = 3). (G) Quantification of lung tissue‐resident bacteria at 24 h.p.i. (*n* = 6). (H) Representative fluorescence images and quantitative analysis of mCherry‐labelled *S. aureus* (red) in cytokeratin 7^+^ LECs (green) in lung tissue of infected mice from different groups at 24 h.p.i. (*n* = 6). Scale bar, 25 µm. (I) Body weight monitoring of sublethally infected mice (1 × 10^8^ CFU *S. aureus*) in different groups (*n* = 6). (J) Survival analysis of lethally infected mice (6 × 10^8^ CFU *S. aureus*) in different groups (*n* = 10). (K, L) H&E staining and histopathological score of lung (K) and spleen tissue (L) in infected mice from different groups at 24 h or 5 days post‐infection (*n* = 6). Scale bar, 100 µm (low‐magnification) and 25 µm (high‐magnification) (K); 200 µm (low‐magnification) and 100 µm (high‐magnification) (L). *SA* denotes *Staphylococcus aureus*. LuEVs denote lung tissue‐derived extracellular vesicles. Data are presented as mean ± SD. Statistical significance was assessed by one‐way ANOVA with Tukey's post hoc test (G–I, K, L) and log‐rank test (J). *P < 0.05, **P < 0.01.

To clarify the role of LuEVs in bacterial internalization by LECs and inflammation regulation during *S. aureus* infection, EVs were isolated from lung tissues of phosphate‐buffered saline (PBS)/*S. aureus‐*treated mice for detailed analysis (Figure 2A) (Crescitelli et al. 2021). Transmission electron microscopy (TEM) revealed the typical cup‐shaped morphology of lipid bilayer‐enclosed vesicles (Figure 2B), while nanoparticle tracking analysis (NTA) yielded a particle size distribution curve with a peak at approximately 160 nm (Figure 2C). Immunoblotting demonstrated positive expression of EV markers, including CD9, CD63, TSG101 and Alix, and negative expression of the endoplasmic reticulum marker calnexin in both kinds of EVs (Figure 2D). These results confirmed the successful isolation of EVs from lungs with no changes in fundamental physical characteristics caused by infection.

Next, to investigate the specific role of EVs in bacterial internalization by LECs, we performed a rescue experiment by re‐supplementing LuEVs isolated from infected mice into GW4869‐pretreated infected mice. Briefly, a mixture of LuEVs and *S. aureus* (LuEVs + *SA*) was intratracheally instilled into mice to assess whether exogenous LuEVs could reverse the inflammatory damage caused by EV inhibition during bacterial challenge (Figure 2E).

Ex vivo imaging and bacterial quantification of lung tissues revealed that LuEVs re‐supplementation markedly led to a striking reversal in the reduced *S. aureus* colonization levels induced by EV inhibition (Figure 2F,G). Immunofluorescence staining also revealed a 4.9‐fold increase of intracellular bacterial load within LECs after LuEVs treatment compared to GW4869‐pretreated infected mice (Figure 2H). Body weight monitoring further verified that the re‐supplementation of LuEVs partially alleviated the weight loss associated with the severe infection caused by EV inhibition at 2 d.p.i. (Figure 2I). Notably, re‐supplementation of LuEVs significantly improved the survival rate of the EVs‐depleted infected mice from 0% to 90% by 60 h.p.i. (Figure 2J). Histological analysis showed that LuEVs treatment substantially mitigated severe inflammatory damage induced by EV inhibition, as evidenced by reduced tissue destruction and inflammatory cell infiltration in the lungs and spleens (Figure 2K,L; Figure S3A–C). In addition, histopathological examination of deceased mice indicated that extremely severe inflammatory infiltration led to almost no visible alveolar cavity in the lungs (Figure S4), confirming that mortality resulted from severe pneumonia rather than sepsis.

To further define the origin of LuEVs, their surface markers were analysed. The majority of LuEVs (73.3%) were found to be derived from epithelial cell (EpCAM^+^), whereas immune cell‐derived EVs (LY6G^+^: 7.22%; F4/80^+^: 3.82%) constituted a minor fraction (Figure S5A). To functionally validate this, EV release from LECs was specifically inhibited via Rab27a knockdown in LECs (Figure S5B) (Hou et al. 2024; Wang et al. 2022), which resulted in a marked reduction of overall EV release and the proportion of EpCAM‐positive EVs within LuEVs (Figure S5C,D). Consequently, specific inhibition of LECs‐EVs significantly reduced bacterial colonization levels within LECs (Figure S5E), and the ability of LuEVs to promote bacterial internalization was reduced (Figure S5F). These results confirm that LECs are the primary source of LuEVs, and that these epithelial‐derived EVs are responsible for facilitating bacterial internalization during infection.

**FIGURE 3:**
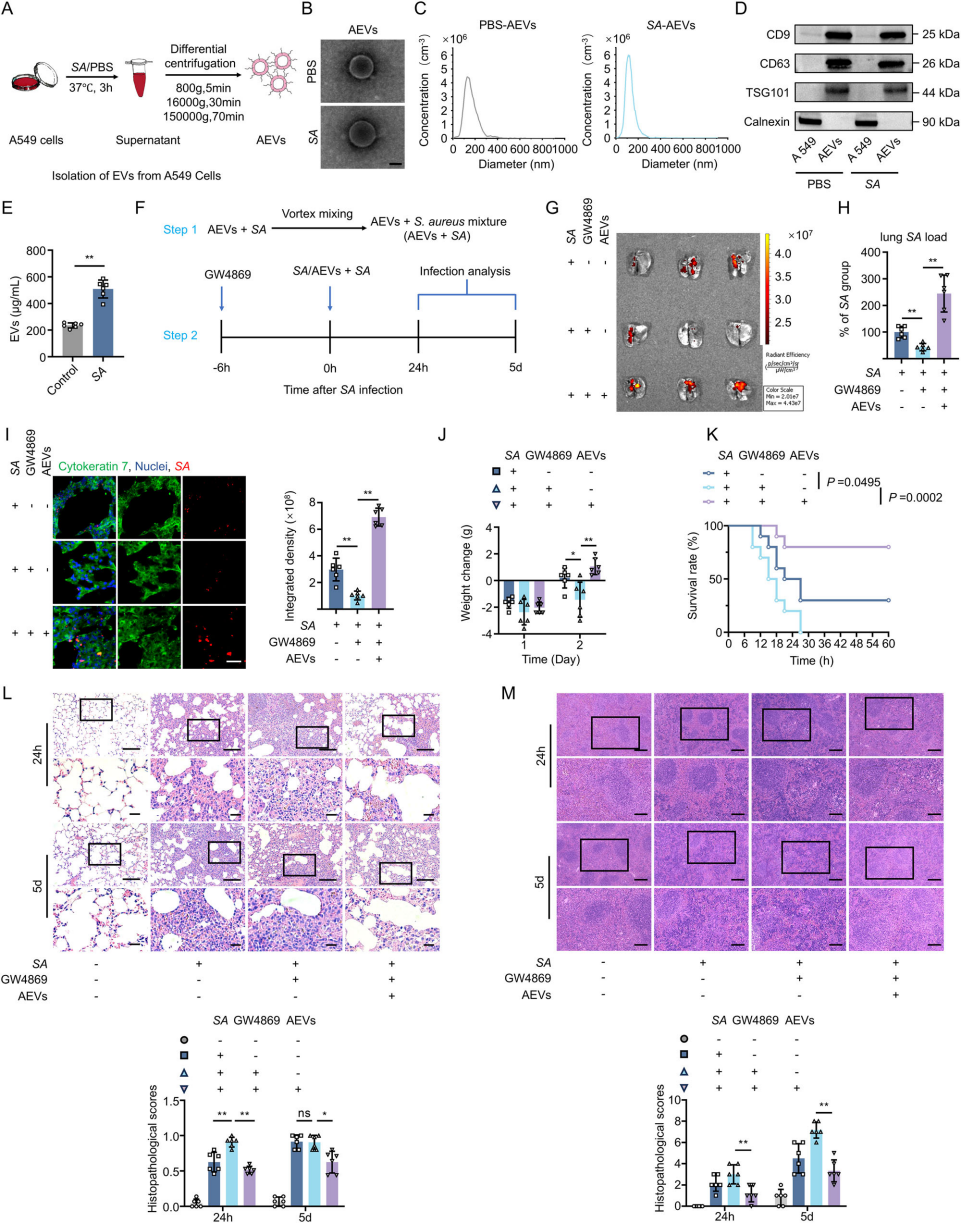
A549‐derived EVs‐mediated *S. aureus* cloaking alleviates pulmonary inflammation and mortality. (A) Schematic diagram of EVs isolation from A549 cell. (B) TEM images of EVs from uninfected A549 cells (PBS‐AEVs) and *S. aureus*‐infected A549 cells (*SA*‐AEVs). Scale bar, 200 nm. (C) Size distribution curve of PBS‐AEVs and *SA*‐AEVs by NTA. (D) Western blot analysis of EVs markers. (E) Quantification of the EV levels released by A549 cells after *S. aureus* infection (*n* = 6). (F) Schematic diagram of AEVs + *SA* mixture (AEVs+*SA*) preparation and in vivo application in mouse infection studies. G) Representative biodistribution of mCherry‐labelled *S. aureus* in mouse lung tissue at 24 h.p.i. (*n* = 3). (H) Quantification of lung tissue‐resident bacteria at 24 h.p.i. (*n* = 6). (I) Representative fluorescence images and quantitative analysis of *S. aureus* (red) level in cytokeratin 7^+^ LECs (green) in lung tissue of infected mice from different groups at 24 h.p.i. (*n* = 6). Scale bar, 25 µm. (J) Body weight monitoring of sublethally infected mice (1 × 10^8^ CFU *S. aureus*) in different groups (*n* = 6). (K) Survival analysis of lethally infected mice (6 × 10^8^ CFU *S. aureus*) in different groups (*n* = 10). (L, M) H&E staining and histological score of lung (L) and spleen tissue (M) in infected mice from different groups at 24 h or 5 days post‐infection (*n* = 6). Scale bar, 100 µm (low‐magnification) and 25 µm (high‐magnification) (L); 200 µm (low‐magnification) and 100 µm (high‐magnification) (M). *SA* denotes *Staphylococcus aureus*. AEVs denote A549 cell‐derived extracellular vesicles. Data are presented as mean ± SD. Statistical significance was assessed by one‐way ANOVA with Tukey's post hoc test (E, H–J, L, M) and log‐rank test (K). *P < 0.05, **P < 0.01.

To corroborate the conclusion that EVs released by LECs facilitate bacterial internalization, A549 cells, a classical LEC line (Ding et al. 2024; Keller et al. 2020), were utilized for in vitro validation. Firstly, EVs derived from A549 cells (AEVs) under various conditions were isolated and further investigated (Figure 3A). The AEVs showed identical physical characteristics to LuEVs (Figure 3B–D), and were released at higher concentrations following *S. aureus* infection (Figure 3E). To clarify the role of AEVs in internalization of *S. aureus* by LECs, *S. aureus* was similarly pre‐mixed with EVs derived from *S. aureus*‐infected A549 cells (AEVs *+ SA*) before lung infection in EV‐depleted mice by GW4869 (Figure 3F). The re‐supplementation of AEVs replicated the effects of LuEVs, increasing bacteria colonization levels in GW4869‐pretreated infected mice while simultaneously improving survival outcomes and reducing inflammatory tissue damage (Figure 3G–M; Figure S6A–C). Collectively, these data demonstrate that LECs mediate bacterial cloaking via EVs release during *S. aureus* infection, thereby protecting the host from excessive inflammatory damage.

### 2.3 Lung Epithelial Cell‐EVs Mediate *S. aureus* Cloaking Through Receptor‐Dependent Binding

**FIGURE 4:**
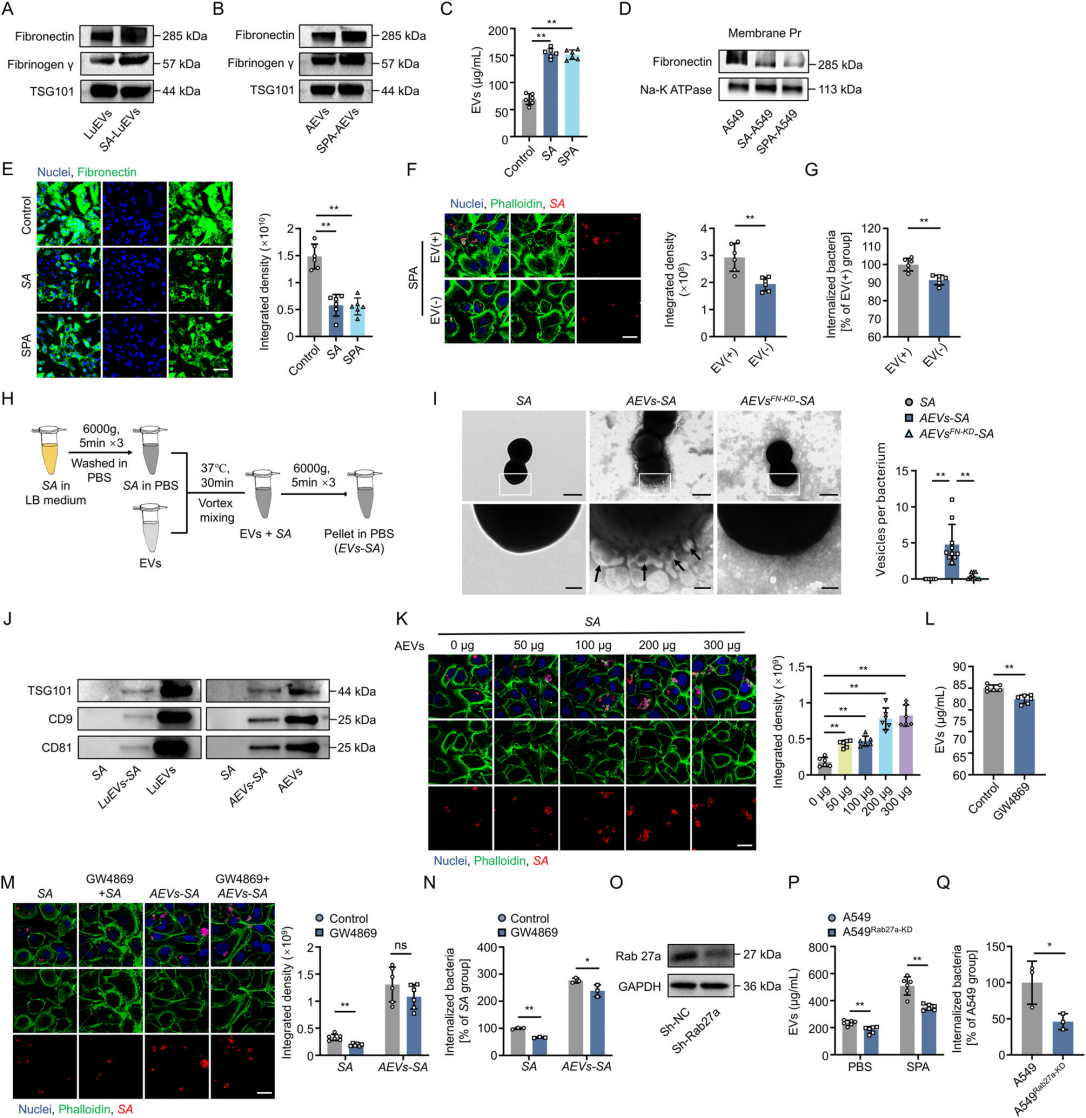
*EV*‐*S. aureus* complex formed by receptor‐mediated binding facilitates bacteria cloaking. (A) Western blot analysis of receptors in LuEVs. (B) Western blot analysis of receptors in AEVs. (C) Comparison of EV concentration in the supernatant of A549 cells before and after *S. aureus* infection or SPA stimulation. (D) Western blot analysis of receptors on the cell membrane. (E) Representative immunofluorescence images and quantitative analysis of fibronectin (green) in A549 cells across different groups. Scale bar, 50 µm (*n* = 6). (F, G) Representative immunofluorescence analysis (F) and intracellular bacterial quantification (G) of *S. aureus* internalization after EV removal from the supernatant of SPA‐stimulated A549 cells (*n* = 6). Scale bar, 25 µm. (H) Schematic diagram of *EVs‐SA* complex (*EVs‐SA*) preparation. (I) TEM images and quantitative analysis of *EVs‐S. aureus* interaction. Black spherical structures are *S. aureus*. The black arrows indicate EVs bound to the bacterial surface. Scale bar, 500 nm (low‐magnification) and 100 nm (high‐magnification). (J) Western blot analysis of TSG101, CD9 and CD81 on *S. aureus* pre‐incubated with EVs (*n* = 3). (K) Representative fluorescence images and quantitative analysis of *S. aureus* (red) level in A549 cells infected with *EV*‐*S. aureus* complexes at EV‐gradient concentrations (*n* = 6). Scale bar, 25 µm. (L) Comparison of EV concentration in the supernatant of A549 cells before and after GW4869 treatment (*n* = 6). (M) Representative fluorescence images and quantitative analysis of intracellular *S. aureus* (red) level in A549 cells across treatment groups (*n* = 6). Scale bar, 25 µm. (N) Quantification of intracellular *S. aureus* level in A549 cells across treatment groups post‐infection (*n* = 3). (O) Western blot analysis of Rab27a in A549 cells transfected with Rab27a shRNA. (P) EV release level from Rab27a‐KD A549 cells under basal and SPA‐stimulated conditions (*n* = 6). (Q) Comparison of *S. aureus* internalization level in A549 cells with and without Rab27a knockdown (*n* = 3). *SA* denotes *Staphylococcus aureus*. AEVs denote A549 cell‐derived extracellular vesicles. LuEVs denote lung tissue‐derived extracellular vesicles. *AEVs‐SA* indicates *AEVs‐S. aureus* complexes. AEVs^FN‐KD^ denotes EVs derived from FN‐knockdown A549 cells. Data are presented as mean ± SD. Statistical significance was assessed by one‐way ANOVA with Tukey's post hoc test. *P < 0.05, **P < 0.01.

*S. aureus* employs a series of surface adhesins, such as fibronectin‐binding protein (FNBP), von Willebrand factor‐binding protein (vWbp) and clumping factor A (ClfA), to mediate adhesion and invasion to host cell (Liesenborghs et al. 2019). EVs are cell‐derived membrane‐bound products expressing surface receptors similar to those of their parental cells. Previous research has demonstrated that EVs containing ACE2 exhibited potent virus‐trapping efficacy in vivo by effectively binding to the spike (S) protein on the surface of SARS‐CoV‐2 (Cocozza et al. 2020). Given these findings, it seems reasonable to highly suspect that the surface of LEC‐EVs expresses receptors capable of coupling with bacteria. Immunoblotting demonstrated that fibronectin (FN) and fibrinogen γ, critical host receptors for *S. aureus* adhesion via FNBPs and ClfA, are expressed on LuEVs and AEVs (Figure 4A,B). Specifically, *S. aureus* infection significantly increased the enrichment of these receptors on LuEVs (Figure 4A). In addition, Staphylococcal protein A (SPA) secreted by *S. aureus* also upregulated FN and fibrinogen γ expression on AEVs (Figure 4B). This indicates that both bacterial infection and specific virulence factor exposure enhance the enrichment of these adhesion receptors on EVs. Given the greater enrichment of fibronectin compared to fibrinogen γ on EVs, fibronectin was selected for further functional validation. Intriguingly, treatment of *S. aureus* or SPA triggered a host defense response characterized by increased EV secretion (Figure 4C), coupled with strategic downregulation of surface fibronectin on A549 cells (Figure 4D,E), which emphasize EVs as functional vehicles for cells to actively expel and neutralize bacteria upon infection. Hence, the depletion of SPA‐induced EVs (SPA‐AEVs) by refreshing the medium also significantly reduced intracellular *S. aureus* loads (Figure 4F,G). These findings suggest that LECs release EVs with upregulated receptor expression upon bacterial stimulation, which may function as mediators to facilitate the interaction with bacteria through receptor‐mediated binding.

Next, direct binding of AEVs to *S. aureus* (experimental design shown in Figure 4H) was confirmed through multiple approaches. Ultrastructural evidence from TEM showed the physical interactions between EVs with bacteria (Figure 4I), while 3D reconstruction of fluorescence imaging further visualized their spatial association in vitro (Figure S7). Besides, immunoblotting confirmed the existence of host‐derived CD9, CD81 and TSG101 on different EVs‐pretreated bacteria pellets (Figure 4J). These findings strongly validate that LEC‐EVs actively adhere to bacterial surfaces to form complex structures via their surface receptors. To further clarify the specific role of FN in EV‐bacteria interactions, FN expression was knocked down in A549 cells via lentiviral‐mediated RNA interference (Figure S8A). The FN‐KD A549 cells released AEVs with reduced surface FN level (AEVs^FN‑KD^) (Figure S8B). Importantly, the adhesion level of AEVs^FN‑KD^ to *S. aureus* was significantly reduced evidenced by TEM and immunoblotting (Figure 4I; Figure S8C).

More detailed functional assays were specifically conducted to clarify the role and interaction mechanisms of the *EVs‐S. aureus* complex (*EVs‐SA*) in vitro. It was initially confirmed that neither EVs derived from different sources nor GW4869 administration exerted detrimental effects on the proliferation kinetics of *S. aureus* (Figure S9A). Additionally, the infection conditions for cell experiments were optimized to ensure the relatively high cell viability without a negative effect on the observation of bacterial adhesion and colonization (Figure S9B). Furthermore, lysostaphin effectively eliminated extracellular free bacteria (Figure S9C). On this basis, the *AEVs‐S. aureus* complex formed by pre‐incubation of *S. aureus* with AEVs significantly increased intracellular bacterial loads in A549 cells in a concentration‐dependent manner, correlating with AEVs uptake efficiency (Figure 4K; Figure S9D,E). Furthermore, GW4869‐mediated pharmacological blockade of EV biogenesis in A549 cells markedly reduced bacterial cloaking, whereas the ability of preformed *AEVs‐S. aureus* complexes to promote *S. aureus* cloaking remained largely unaffected by GW4869 treatment (Figure 4L–N). Consistent with this finding, Rab27a knockdown‐mediated EVs inhibition similarly suppressed *S. aureus* cloaking in A549 cells but did not impair the effect of preformed *AEVs‐S. aureus* complexes, yielding results comparable to pharmacological blockade (Figure 4O–Q). In addition, AEVs^FN‑KD^ exhibited a diminished ability to promote bacterial internalization into A549 cells compared with control AEVs (Figure S8D). These findings suggest that EVs released by LECs during bacterial infection play a critical role in facilitating bacterial cloaking through surface receptor‐mediated binding.

Notably, SPA‐AEVs exerted similar effects in mediating bacterial cloaking in LECs, as demonstrated by bacterial quantification assays at both the population and single‐cell levels (Figure S9F,G). Immunofluorescence analysis revealed that while AEVs did not affect macrophage phagocytosis (Figure S9H,I), LuEVs modestly increased the uptake of *S. aureus* by lung tissue‐resident macrophages (Figure S9J). Notably, in the *LuEVs‐S. aureus* complex‐treated lung tissue, EpCAM^+^ epithelial cells constituted 69.5% of all *S. aureus*‐positive cells, compared to 17.8% for F4/80^+^ macrophages (Figure S9K), confirming the dominant contribution of epithelial cells over macrophages to bacterial cloaking in vivo.

The observed EV‐mediated bacterial cloaking mechanism exhibited broad relevance across some other cell types. SPA also significantly increased EV secretion from human umbilical vein endothelial cells (HUVECs) (Figure S10A). Meanwhile, EVs generated by HUVEC following SPA stimulation (SPA‐HEVs) also exhibited binding capacity to *S. aureus* (Figure S10B). Additionally, bacterial quantitation and immunofluorescence assays collectively demonstrated that SPA‐HEVs significantly enhanced the internalization of *S. aureus* by HUVECs (Figure S10C–E). The mechanism convergence observed in these experiments highlights that SPA triggers pleiotropic cellular responses, inducing the secretion of EVs with *S. aureus*‐specific binding ability. This EV‐mediated process ultimately facilitates bacterial cloaking within host cells, suggesting a conserved strategy across multiple cell types.

### 2.4 *EV‐S. aureus* Complexes Hijack Caveolin‐Dependent Endocytosis for Bacteria Cloaking

**FIGURE 5:**
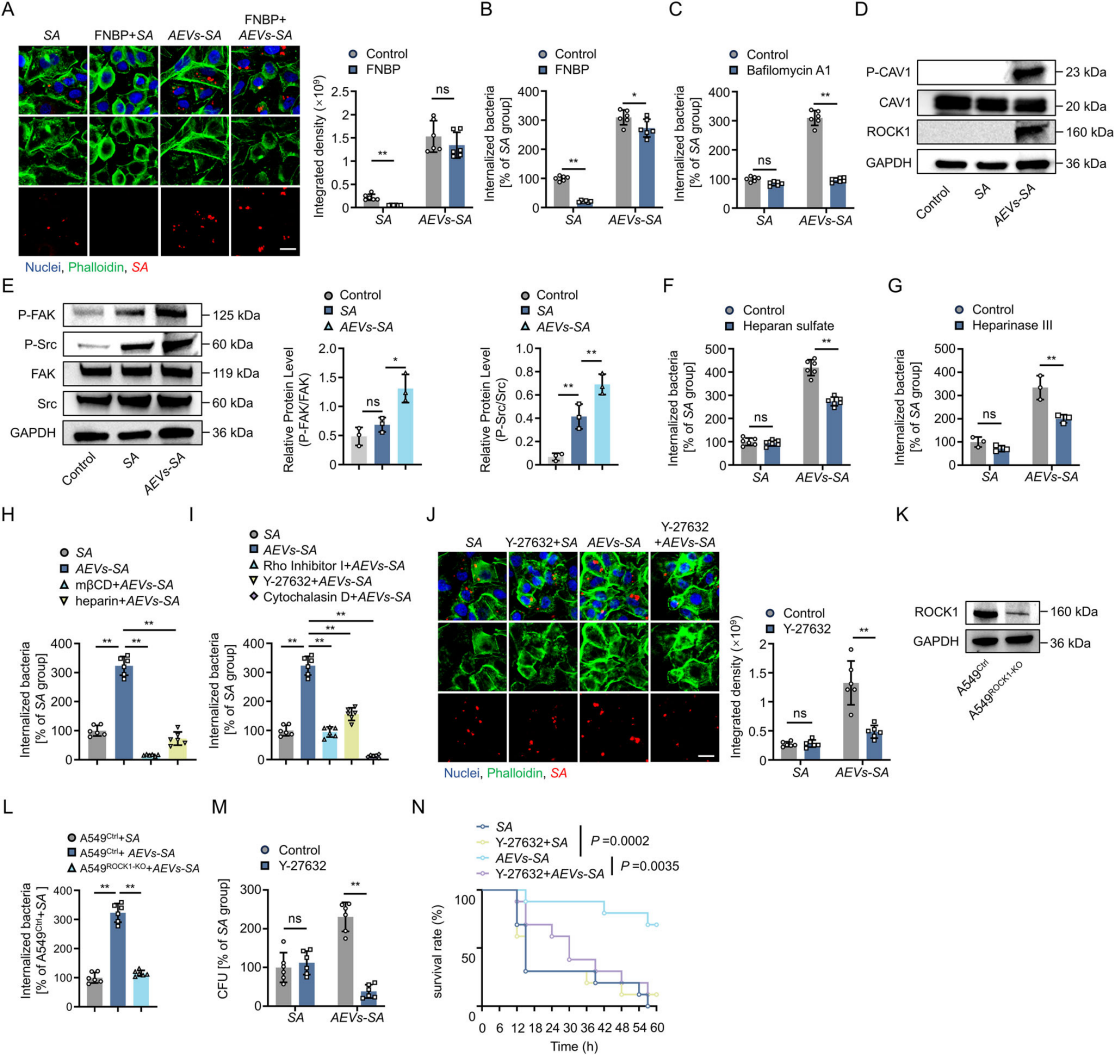
The internalization of *AEVs‐S. aureus* complexes by host cells through the RhoA‐ROCK1‐actin‐driven endocytosis pathway. (A) Representative fluorescence images and quantitative analysis of intracellular *S. aureus* (red) level in A549 cells across treatment groups (*n* = 6). Scale bar, 25 µm. (B) Quantification of intracellular *S. aureus* level in A549 cells across defined treatment groups post‐infection (*n* = 6). (C) Quantification of *S. aureus* level in A549 cells with or without bafilomycin A1 treatment across *SA* and *AEVs‐SA* groups (*n* = 6). (D) Western blot analysis of phospho‐CAV‐1, CAV‐1 and ROCK1 in A549 cell lysates collected at 3 h.p.i. across experimental groups. (E) Western blot analysis of phospho‐FAK, phospho‐Src, FAK and Src in A549 cell lysates at 3 h.p.i. (*n* = 3). (F) Quantification of intracellular *S. aureus* level in *SA* and *AEVs‐SA* groups after cell surface HSPG antagonism via heparan sulfate (*n* = 6). (G) Quantification of intracellular *S. aureus* level in *SA* and *AEVs‐SA* groups after HSPG enzymatic removal by Heparinase III (*n* = 6). (H) Quantification of intracellular *S. aureus* level in *AEVs‐SA* groups after cell surface cholesterol removal with mβCD and lipid removal with heparin (*n* = 6). (I) Comparison of intracellular bacterial load in A549 cells treated with *AEVs‐SA* following pharmacological inhibition of RhoA, ROCK1, or actin polymerization (*n* = 6). (J) Representative fluorescence images and quantitative analysis of intracellular *S. aureus* (red) level in Y‐27632‐treated and untreated A549 cells (*n* = 6). Scale bar, 25 µm. (K) Western blot analysis of ROCK1 in control and knockout A549 cells. (L) Quantification of intracellular *S. aureus* level in *EVs‐SA* group before and after ROCK1 knockout (*n* = 6). (M) Quantification of lung tissue‐resident bacteria at 24 h.p.i., following ROCK1 inhibition (*n* = 6). (N) Survival analysis of lethally infected mice (6 × 10^8^ CFU *S. aureus*) in different groups (*n* = 10). *SA* denotes *Staphylococcus aureus*. *AEVs‐SA* indicates *AEVs‐S. aureus* complexes. Data are presented as mean ± SD. Statistical significance was assessed by one‐way ANOVA with Tukey's post hoc test (A–M) and log‐rank test (N). *P < 0.05, **P < 0.01.

After EVs bind to bacteria and form complexes, the mechanism underlying cellular uptake and internalization of these complexes warrants further investigation. *S. aureus* invades epithelial cells through a well‐characterized mechanism involving the formation of fibronectin bridges between bacterial FNBPs and host integrin α5β1 receptors, which could be effectively inhibited by soluble recombinant fibronectin‐binding domains (D1‐D4) of FNBP (Sinha et al. 1999). Recombinant fibronectin‐binding protein (FNBP) D1‐D4 peptides significantly inhibited the internalization of free *S. aureus*, but showed limited efficacy against the internalization of *AEVs‐S. aureus* complexes (Figure 5A). Lung bacterial quantification revealed that FNBP‐mediated inhibition efficacy of intracellular colonization against *AEVs‐S. aureus* complexes was markedly lower than that against free *S. aureus* under identical experimental conditions (Figure 5B). This finding provides compelling evidence that *EVs‐S. aureus* complexes employ a distinct internalization pathway into host cells, fundamentally diverging from canonical bacteria internalization paradigms. Given that the presence and composition of EVs on the surface of *EVs‐S. aureus* complexes, we hypothesize that these complexes enter host cells through an endocytic pathway primarily dependent on EVs. Subsequently, bafilomycin A1, a potent inhibitor of vacuolar ATPase known for its efficacy in suppressing EV endocytosis (Mulcahy et al. 2014), effectively suppressed intracellular bacterial colonization within A549 cells treated with *AEVs‐S. aureus* complexes (Figure 5C). To further delineate the specific endocytic pathway, the effects of pathway‐specific inhibitors were tested. Notably, Nystatin, which disrupts caveolin‐dependent endocytosis (Ferreira et al. 2025), markedly inhibited *AEVs‐S. aureus* complex internalization (Figure S11). In contrast, neither CPZ nor amiloride exerted obvious inhibitory effects (Figure S11), ruling out major contributions from clathrin‐dependent or macropinocytic mechanisms. These results confirm that *EVs‐S. aureus* complexes internalization primarily occurs via the caveolin‐dependent endocytic pathway.

Growing evidence suggests that caveolin‐1 (CAV1) and Ras homolog family member A (RhoA) play critical roles in caveolin‐dependent endocytosis (Jong et al. 2020; Kooijmans et al. 2021). RhoA, a GTPase regulating actin cytoskeleton activated by CAV1, drives cell morphology, migration and vesicular trafficking. CAV1 phosphorylation induces Rho activation, which subsequently activates ROCK, ultimately resulting in the phosphorylation of focal adhesion kinase (FAK) and Src (Joshi et al. 2008; Liu et al. 2018). Infection with *AEVs‐S. aureus* complexes triggered robust CAV1 phosphorylation and ROCK1 expression (Figure 5D), initiating a signalling cascade that activates phosphorylation of FAK and Src (Figure 5E). Since CAV1‐mediated lipid raft‐dependent endocytosis critically depends on heparan sulfate proteoglycans (HSPG) on the cell surface (Rai and Johnson 2019), either competitive blockade of HSPG binding sites by exogenous heparan sulfate or enzymatic removal of cell surface HSPGs by heparinase III significantly weakened the internalization of *AEVs‐S. aureus* complexes, with no effect on the internalization of free *S. aureus* (Figure 5F,G).

Given the essential role of cholesterol in maintaining lipid raft integrity within caveolae (Kooijmans et al. 2021) and the sensitivity of caveolin‐dependent endocytosis to cholesterol depletion (Rai and Johnson 2019), the internalization mechanism of *AEVs‐S. aureus* complexes requires intact lipid raft microdomains. Thus, both MβCD‐mediated cholesterol depletion and heparin‐mediated disruption of lipid raft‐HSPG interactions significantly reduced *AEVs‐S. aureus* complexes internalization in A549 cells (Figure 5H). Pharmacological intervention at multiple nodes in the signalling pathway targeting RhoA (Rho inhibitor I) and ROCK1 (Y‐27632) consistently inhibited the internalization of *AEVs‐S. aureus* complexes into host cells (Figure 5I,J). Cytochalasin D also inhibited the internalization of the *AEVs‐S. aureus* complexes and weakened the bacteria cloaking effect by disrupting the actin skeleton (Figure 5I). The essential role of ROCK1 was further established in ROCK1‐knockout A549 cells (A549^ROCK1‐KO^), where its ablation affected the EVs‐mediated bacterial cloaking effect (Figure 5K,L). Consistent with in vitro observations, pharmacological inhibition of ROCK1 by Y‐27632 in vivo significantly reduced lung tissue‐resident bacteria and worsened infection outcomes (Figure 5M,N).

These results collectively define a non‐canonical pathway that the formation of *EV*‐*S. aureus* complexes promotes bacterial cloaking through the RhoA‐ROCK1‐actin‐driven endocytosis pathway, distinct from the well‐characterized bacterial internalization mechanism. While this EV‐mediated pathway enhances early‐stage bacterial cloaking, beneficial to moderate inflammatory responses, its long‐term consequences for disease progression remain to be fully elucidated.

### 2.5 EVs‐Mediated Intracellular Colonization of *S. aureus* Drives Durable Chronic Infection

**FIGURE 6:**
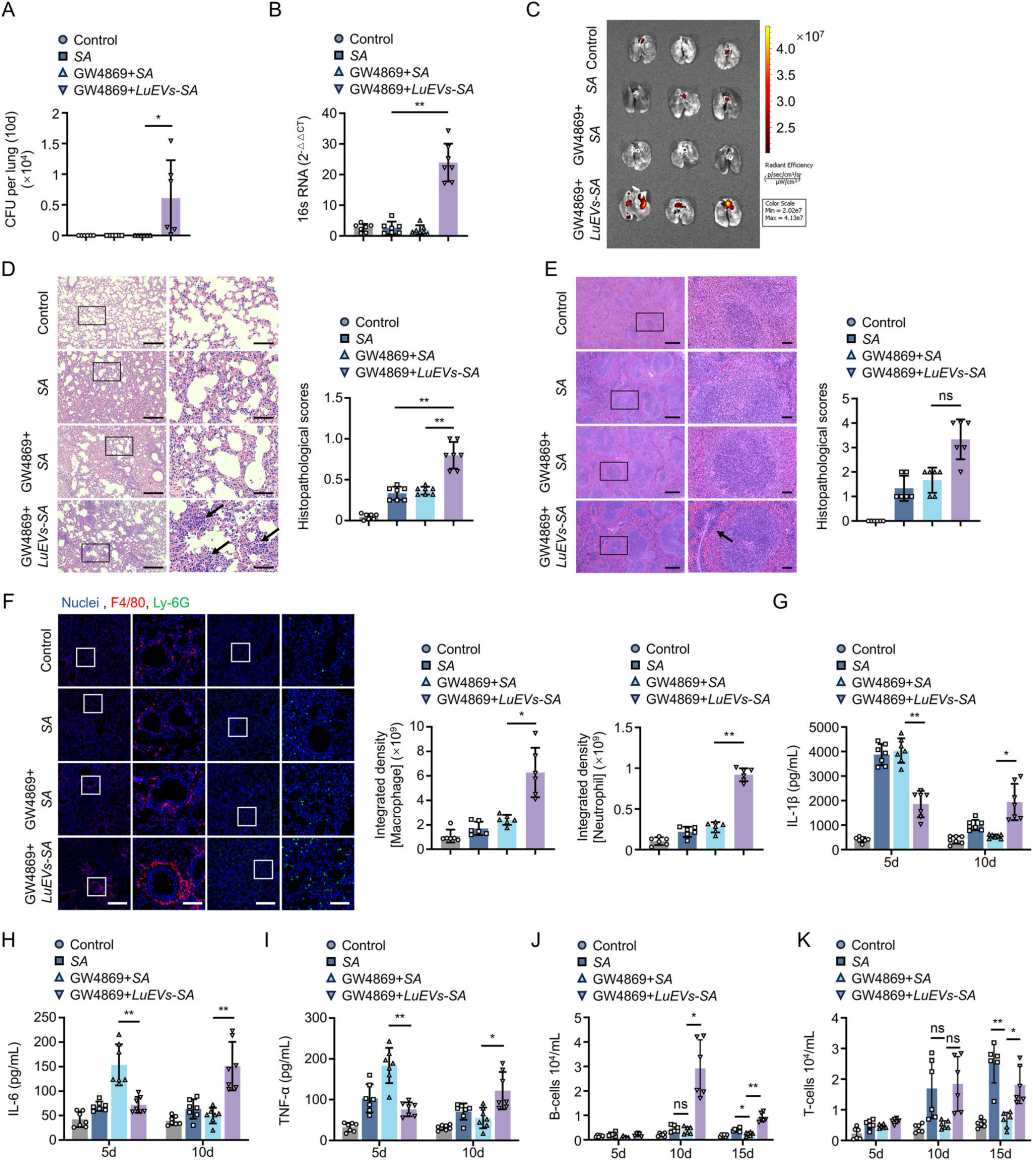
EV‐mediated intracellular colonization of *S. aureus* underlies chronic infection pathogenesis. (A) Quantification of lung tissue‐resident bacteria at 10 d.p.i. (*n* = 6). (B) Quantification of *S. aureus* 16S rRNA in pulmonary tissue at 10 d.p.i. (*n* = 6). (C) Representative biodistribution of mCherry‐labelled *S. aureus* in mouse lung tissue at 10 d.p.i. (*n* = 3). (D, E) H&E staining and histopathological scores for lungs (D) and spleens (E) at 10 d.p.i. (*n* = 6). Scale bar, 200 µm (low‐magnification) and 50 µm (high‐magnification) (D); 300 µm (low‐magnification) and 50 µm (high‐magnification) (E). Black arrows indicate pulmonary abscesses in 6D and splenic inflammatory cell infiltration in 6E. (F) Representative fluorescence images and quantitative analysis of F4/80^+^ macrophages (red) and Ly‐6G^+^ neutrophils (green) in lung tissue at 10 d.p.i. (*n* = 6). Scale bar, 400 µm (low‐magnification) and 100 µm (high‐magnification). (G–I) ELISA analysis of IL‐1β (G), IL‐6 (H) and TNF‐α (I) in pulmonary homogenates at 5 and 10 d.p.i. (*n* = 7). (J, K) Flow cytometry of B cells (J) and T cells (K) in bronchoalveolar lavage fluid at 5, 10 and 15 d.p.i. (*n* = 6). *SA* denotes *Staphylococcus aureus*. LuEVs denote lung tissue‐derived extracellular vesicles. *LuEVs‐SA* indicates *LuEVs‐S. aureus* complexes. Data are presented as mean ± SD. Statistical significance was assessed by one‐way ANOVA with Tukey's post hoc test. *P < 0.05, **P < 0.01.

Decades of research have solidified the concept that internalization of bacteria by non‐professional phagocytes creates protective intracellular compartments, thereby facilitating immune evasion and sustaining persistent infection (Bertuzzi et al. 2019; Gal‐Mor 2019; Sachse et al. 2010). To determine the long‐term impact of *S. aureus* cloaking by LECs at the early stage, the lung bacterial load in the infected mice were examined at 10 d.p.i. using bacterial colony counting, *S. aureus* 16S rRNA quantification and ex vivo imaging. Simple *S. aureus* infection showed nearly complete bacterial clearance by 10 d.p.i. regardless of GW4869 pretreatment, *LuEVs‐S. aureus* complexes led to persistence of *S. aureus* infection in lung tissue until 10 d.p.i. (Figure 6A–C), accompanied by severe abscess formation in lungs and sustained inflammatory cell infiltration in both lungs and spleen (Figure 6D–F). This persistent bacterial reservoir triggered a unique cytokine profile characterized by delayed elevation of IL‐1β, IL‐6 and TNF‐α levels at 10 d.p.i. (Figure 6G–I), in sharp contrast with the transient cytokine spikes observed in acute infections. Chronic pulmonary infection is pathologically defined by the sustained activation of adaptive immunity, particularly through the infiltration of B and T lymphocytes (Frija‐Masson et al. 2017). Flow cytometry analysis of bronchoalveolar lavage fluid (BALF) demonstrated that while GW4869 attenuated lymphocyte recruitment in *S. aureus*‐only infections, *LuEVs‐S. aureus* complexes maintained elevated B and T cell populations at 15 d.p.i. (Figure 6J,K). These findings collectively demonstrate that EVs‐mediated intracellular bacterial cloaking induces persistent infection and chronic inflammation in lung tissue.

In conclusion, our findings reveal a previously unrecognized mechanism by which non‐professional phagocytic cells employ bacterial cloaking as a strategy to resist acute infection and secure host survival. In this paradigm, bacteria cloaking is coordinated through EV‐orchestrated endocytosis pathway within LECs during *S. aureus* infection, which serves to reduce the free bacteria load and mitigate acute‐phase inflammatory pathology. Paradoxically, this interaction allows the bacteria exploit this cloaking mechanism to form persistent intracellular colonization, thereby evading host immune surveillance and contributing to chronic infection. Therefore, this EV‐mediated bacteria cloaking mechanism represents an evolutionary trade‐off in host‐pathogen interaction, enabling survival during acute infection at the expense of latent intracellular bacterial infection.

## 3 Discussion

Overwhelming bacterial challenge represents a dominating driver of lethal inflammation during pulmonary infection (Vaughn et al. 2024). Beyond the immune system's direct antimicrobial actions, emerging evidence suggests that hosts employ pathogen cloaking strategies to achieve immunological transition, thereby avoiding collateral tissue damage from direct confrontation (Neupane et al. 2020). This study establishes LECs as pivotal mediators of an active inflammation‐regulation mechanism against *S. aureus*‐induced pneumonia. During acute infection, LEC‐derived EVs act as decoys to trap bacteria, thereby enhancing host cell internalization and sequestration of pathogens. This cloaking process effectively attenuates free bacterial burden, a primary driver of inflammatory responses and lung tissue destruction, ultimately improving survival rates in infected mice models. While pathogen cloaking by professional phagocytes is well‐documented, its efficacy is constrained by the limited abundance of tissue‐resident macrophages in pulmonary tissue (Neupane et al. 2020). Evolutionary considerations suggest that LECs, as primary constituents of lung epithelium exposed to persistent environmental challenges (Paulsson and Riesbeck 2018), must possess intrinsic protective mechanisms. This study redefines the pathogen cloaking capacity of LECs as non‐professional phagocytes, demonstrating their reliance on EV‐mediated extracellular pathogen cloaking rather than direct cellular internalization. This work updates the conventional paradigm that positions LECs solely as passive barriers or amplifiers of inflammatory signalling, instead establishing their critical contribution to early‐phase immune regulation through EV‐dependent bacterial cloaking mechanisms.

The molecular mechanism elucidated in this study demonstrates a dual regulatory paradigm of hEVs in bacterial pathogenesis. Contrary to previous unidimensional views of EVs as either host‐derived pathogen defense systems, as exemplified by iron‐sequestration mechanisms (Kuang et al. 2023), or pathogen‐exploited invasion tools, particularly for virulence factor delivery (Abrami et al. 2013), LEC‐derived EVs were found to engage in host‐pathogen crosstalk through non‐canonical mechanisms. As a typical extracellular pathogen, *S. aureus* induces acute pulmonary infection characterized by neutrophil‐dominant infiltration within 7 days post‐infection (d.p.i.) (Frija‐Masson et al. 2017). Free *S. aureus* is rapidly cleared by the host immune system and does not persist beyond 48–72 h.p.i. (Kebaier et al. 2012). To establish chronic *S. aureus* infection models, additional interventions are required, such as agarose bead‐embedded bacteria, to evade rapid immune clearance and circumvent the high mortality associated with bolus infection, with such chronic infections persisting for 7–14 d.p.i. and marked by a prominent shift toward lymphocyte accumulation in lung tissues (Frija‐Masson et al. 2017). Aligning with these established infection paradigms, this study reveals evolutionarily conserved immunomodulatory trade‐offs in host‐microbe interactions through the context‐dependent dual role of EVs. During acute infection, spatial confinement via EV‐mediated cloaking significantly diminishes extracellular pathogen exposure, representing a host‐priority immunoregulatory strategy that attenuates lethal hyperinflammatory responses. Conversely, this mechanism enables pathogens to exploit non‐canonical internalization pathways for intracellular niche formation, constituting a pathogen‐derived immune evasion strategy consistent with the cost‐benefit trade‐off principle (Medzhitov 2021). This bidirectional interaction highlights that hosts may tolerate suboptimal pathogen clearance to gain acute‐phase survival advantages, while pathogens co‐opt hEVs as immunoprivileged carriers for persistent colonization. Collectively, the dual role highlights the complexity of EV‐mediated immunity and underscores the need to dissect context‐specific regulatory mechanisms.

This study provides insights into the clinical conundrum of chronic pneumonia from the perspective of vesicle biology, laying a theoretical foundation for developing EV‐pathogen interface‐targeted therapeutic strategies. During the acute phase, augmentation of EV‐mediated bacterial cloaking through exogenous EV administration or RhoA agonist may accelerate bacteria cloaking and mitigate excessive inflammatory responses. In contrast, during chronic stages, interventions should focus on regulating EV biogenesis to prevent EV‐mediated intercellular bacterial transmission. More importantly, nanocarrier‐mediated lysosomal enzyme delivery system should be designed to enhance intracellular bactericidal efficiency. The dual functionality of EVs presents significant therapeutic challenges. Pathogens may exploit hEVs to form invasive vesicle complexes, necessitating the development of discriminative biomarkers to distinguish spatiotemporally specific host‐protective EV subpopulations from pathogen‐hijacked ones. There are also several limitations in this study. Firstly, the potential contribution of EVs released by immune cells to the bacterial cloaking process has not been precisely quantified, and the role of MVs released by *S. aureus* in this context remains to be elucidated. Secondly, the spatiotemporal heterogeneity of LECs at different infection stages remains incompletely characterized, hindering a comprehensive understanding of EV release patterns and function. Additionally, the fate of intracellular bacteria and their crosstalk with host cells require more systematic elucidation. Future research will employ lung organoid cultures focus on analysing the spatiotemporal heterogeneity of LECs after infection, distinguishing host‐protective EVs from pathogen‐hijacked subpopulation, identifying key points for intracellular bacterial eradication, and evaluating the role of immune cell‐derived EVs in this process (Ren et al. 2023; Tavares‐Negrete et al. 2023). These efforts aim to identify precise therapeutic targets for resolving post‐infectious chronic inflammation.

## 4 Methods

### 4.1 Bacterial Strains and Growth Conditions

The mCherry‐labelled *S. aureus* Newman strain (Newman‐mCherry) and GFP‐labelled *S. aureus* Newman strain (Newman‐GFP) were sourced from Hangzhou Biosci Biological Co., Ltd. Bacterial cultures were grown in Luria‐Bertani (LB) broth (10 g/L tryptone, 5 g/L yeast extract, 10 g/L NaCl) supplemented with 10 µg/mL chloramphenicol, with continuous shaking (200 rpm) at 37°C for 16–18 h. Bacterial density was determined by serial dilution plating on LB agar, followed by colony counting after 24‐h incubation at 37°C.

### 4.2 Cell Culture

The A549 cell line (Procell, CL‐0016) was cultured in Ham's F‐12K medium (Procell, PM150910) supplemented with 10% fetal bovine serum (FBS) and 1% penicillin/streptomycin. ROCK1‐knockout A549 cells (Ubigene, YKO‐H897) were maintained under identical conditions. Bone marrow‐derived macrophages (BMDMs) were isolated from femurs and tibias of C57BL/6J mice. After PBS washing and erythrocyte lysis (Beyotime, C3702), cells were centrifuged (800 × *g*, 5 min) and cultured in DMEM (Gibco, 11965092) with 10% FBS and 20 ng/mL M‐CSF (PeproTech, 315‐02) for 7–8 days to achieve terminal differentiation. Human umbilical vein endothelial cells (HUVECs, Fuheng Biotechnology, FH1122) were grown in DMEM containing 10% FBS and 1% penicillin/streptomycin. All cells were cultured in six‐well plates at 37°C in a humidified 5% CO_2_ atmosphere. The medium was replaced every 48 h, and cells were passaged upon reaching 80%–90% confluency. Before bacterial exposure, cells were washed with PBS and transferred to antibiotic‐free, serum‐reduced medium. Infection protocols used 3‐h exposures at specified MOI: BMDMs (MOI 10), A549/HUVECs (MOI 50). Pharmacological treatments included: Bafilomycin A1 (MedChemExpress, HY‐100558; 10 nM, 30 min), GW4869 (MedChemExpress, HY‐19363; 20 nM, 10 h), Y‐27632 (MedChemExpress, HY‐10071; 1 µM, 30 min), Heparan sulfate (MedChemExpress, HY‐101916; 100 µg/mL, 30 min), Heparin (MedChemExpress, HY‐17567A; 10 µg/mL, 4 h), Cytochalasin D (MedChemExpress, HY‐N6682; 1 µg/mL, 4 h), Chlorpromazine (MedChemExpress, HY‐12708; 10 µg/mL, 30 min), Nystatin (MedChemExpress, HY‐17409; 25 µg/mL, 30 min), Amiloride (MedChemExpress, HY‐B0285; 1 mmol/L, 30 min), Heparinase III (New England Biolabs, P0737S; 10 mU, 30 min), Rho Inhibitor I (Cytoskeleton, CT04; 0.5 µg/mL, 4 h), SPA (Sino Biological, 10600‐P07E; 100 µg/mL, 12 h).

### 4.3 Animal Experiment

Animal experiments received approval from the Animal Management Committee of the Fourth Military Medical University. Female C57BL/6J mice (4–5 weeks old) were housed under controlled conditions (22°C ± 2°C, 40% humidity, 12‐h light/dark cycles). To evaluate EV involvement in *S. aureus* pathogenesis, GW4869 (2.5 µg/g) was administered intratracheally 6 h before pulmonary challenge with 1 × 10^8^ CFU *S. aureus*. Bacterial suspensions were prepared in PBS, while GW4869 was dissolved in DMSO (10 mg/mL stock) and diluted in PBS. Tissues were collected at 24 h/5 days post‐infection for bacterial quantification and histopathology. PBS‐administered mice served as controls. Body weight changes were recorded daily in mice infected with 1 × 10^8^ CFU. Survival rates were monitored at 3‐h intervals for 60 h following challenge with 6 × 10^8^ CFU. For ROCK1 analysis, Y‐27632 (10 µg/g) was administered intratracheally 4 h before infection, with subsequent assessment of pulmonary bacterial loads and survival kinetics.

### 4.4 EV Isolation and Characterization

EVs were isolated from cellular supernatants or serum samples through differential centrifugation. Samples were sequentially centrifuged at 800 × *g* (5 min, 4°C), 16,000 × *g* (30 min, 4°C) and 150,000 × *g* (70 min, 4°C), with intermediate PBS washing steps. Final EV pellets were resuspended in sterile PBS after collection from the ultracentrifugation tube bases. EV concentrations were determined by BCA assay (Beyotime Biotechnology, P0012S) and NTA (Malvern Panalytical, NanoSight NS300). For tracking analysis, EV suspensions were diluted to 10–100 particles/frame. Particle size distributions were quantified using a Nanosight LM10 system (Malvern Panalytical) with ZetaView PMX 120 hardware (Particle Metrix), analysed through ZetaView software (v8.04.02) under standardized optical settings.

### 4.5 Bacterial Load Quantification in Organ Tissues

Lung tissues were aseptically harvested. Prior to homogenization, tracheal instillation of lysostaphin (20 µg/mL) was performed, followed by PBS rinsing. The tissues were then weighed and homogenized in PBS (1 mL per 100 mg tissue). Homogenates were serially diluted (10‐fold increments), plated on LB agar (100 µL aliquots) and aerobically incubated at 37°C for 24 h. Bacterial burden was quantified by CFU enumeration.

### 4.6 Preincubation of *EVs‐S. aureus* Complexes

GFP‐labelled *S. aureus* and mCherry‐labelled *S. aureus* were grown to the logarithmic phase, harvested by centrifugation at 6000 × *g* for 5 min, and washed three times with PBS. The bacterial suspensions were adjusted to 1 × 10^8^ CFU/mL and then incubated with varying concentrations of LuEVs or AEVs. For the infection of 1 × 10^6^ cells in vitro, approximately 5.2 × 10^7^ CFU of *S. aureus* were used, with a corresponding AEVs dose of 100 µg. The mixtures were incubated at 37°C with shaking at 200 rpm for 30 min. For live‐cell super‐resolution imaging, EVs were pre‐stained with PKH67 (Sigma–Aldrich, MINI67) per the manufacturer's instructions before being combined with the bacteria.

### 4.7 Immunofluorescence Staining

Lung tissues were fixed in 4% PFA for 24 h at 4°C, washed with PBS three times (5 min/wash), and cryoprotected in 30% sucrose (w/v) for 24 h before OCT embedding. Then, 10‐µm‐thick sections were cut using a Leica CM1860 cryostat microtome. To eliminate extracellular *S. aureus* contaminants, sections were treated with 20 µg/mL lysostaphin (Sigma–Aldrich, L7386) for 30 min at 37°C. Next, permeabilization was done with 0.1% Triton X‐100 (v/v, Sigma–Aldrich, 93443) for 10 min, followed by blocking with 5% normal goat serum (Boster Biological Technology, AR0009) for 1 h at room temperature. Sections were incubated overnight at 4°C with primary antibodies: anti‐cytokeratin 7 (ABclonal, A4765, 1:200), anti‐F4/80 (Abcam, ab6640, 1:200), anti‐Ly‐6G (BioLegend, 127601, 1:200), and anti‐C Reactive Protein (Servicebio, GB115545, 1:200). After three 5‐min PBS washes, sections were exposed to Alexa Fluor 488/594‐conjugated secondary antibodies (YeaSen, 1:200) for 1 h at 37°C. Nuclear counterstaining was performed with 10 µg/mL Hoechst 33342 (Sigma–Aldrich, ST9H9BC1534E) for 10 min. Finally, immunofluorescence images were captured by CLSM (Nikon), and fluorescence intensity was analysed with ImageJ software.

A549 cells and BMDMs were seeded onto 12 mm glass coverslips in 24‐well culture plates at 5 × 10^4^ cells/well. After experimental interventions, cells were washed with PBS, treated with 20 µg/mL lysostaphin (Sigma, L7386) for 30 min to remove extracellular *S. aureus*, fixed with 4% PFA for 30 min, and blocked with 5% BSA for 1 h at room temperature. Then, cells were incubated overnight at 4°C with Alexa Fluor 488‐conjugated phalloidin (YeaSen, 40735ES75, 1:200) or anti‐fibronectin antibody (Abcam, ab268020, 1:200), followed by secondary antibody incubation. Hoechst staining was applied, and cells were visualized by CLSM.

### 4.8 Super‐Resolution Imaging and 3D Reconstruction of EVs‐Bacterial Complexes

Visualization of *EVs‐S. aureus* complexes was performed by super‐resolution structured illumination microscopy (SR‐SIM). Briefly, mCherry‐expressing *S. aureus* were co‐incubated with PKH67‐labelled EVs, and 5 µL of the mixture was prepared for immediate imaging on a ZEISS Elyra 7 microscope. To ensure EV integrity, imaging was conducted within a 30‐min window post‐sample preparation. Three‐dimensional models were subsequently rendered with Imaris (v10.2.0).

### 4.9 Histopathological Evaluation

Lung and spleen specimens were fixed in 4% PFA at 4°C for 24 h, followed by routine paraffin embedding protocols. Serial 4‐µm sections were cut using a Leica RM2235 rotary microtome and stained with H&E. Histopathological analysis was conducted using a Leica DM4B microscope with LAS X software (v3.7.4). Pulmonary injury severity was assessed via a modified histopathological scoring system (Matute‐Bello et al. 2011). scored from 0 (no injury) to 1 (severe injury). The score incorporated five key parameters: alveolar and interstitial neutrophil density, interstitial neutrophil density, hyaline membrane formation, airspace protein deposition and alveolar septal thickening ratio.

For splenic histological evaluation, a semiquantitative scoring system was applied. Splenic changes were classified based on three criteria: architectural preservation, necrotic cell presence and inflammation extent. These changes were graded as absent (0), slight (1), moderate (2) or pronounced (3).

### 4.10 Western Blot

Tissue samples were lysed on ice for 30 min in Beyotime lysis buffer containing a protease inhibitor. Protein concentrations were determined using the BCA method, and 20 µg of protein was loaded onto an 8%–12% SDS‐polyacrylamide gel. After electrophoresis, proteins were transferred to a PVDF membrane (Merck Millipore) for 2 h. The membrane was blocked with 5% BSA at room temperature for 1 h, then incubated with primary antibodies (detailed below) at 4°C overnight. Following this, the membrane was treated with HRP‐conjugated secondary antibodies at room temperature for 1 h. Protein bands were detected using a chemiluminescence kit (AccuRef Scientific, AP0081S) and visualized with a Tanon 4600 imaging system.

The primary antibodies were sourced as follows: anti‐TSG101 (ab125011), anti‐calnexin (ab22595), anti‐sodium potassium ATPase (ab76020), and anti‐Fibronectin (ab268020) from Abcam; anti‐Fibrinogen γ (D6) (sc‐133226) from Santa Cruz; anti‐Alix (92880), anti‐ROCK1 (4035), anti‐Phospho‐FAK (Tyr397) (3283s), and anti‐Rab27a (69295s) from Cell Signalling Technology; anti‐CD9 (A1703), anti‐CD63 (A5271), anti‐Caveolin‐1 (A22417), anti‐Phospho‐Caveolin‐1‐Y14 (AP0742), anti‐Src (A19119), anti‐Phospho‐Src‐Y419 (AP1027), anti‐FAK (A11131) and anti‐GAPDH (A19056) from ABclonal; anti‐CD9 (HY‐P80610) from MedChemExpress. Secondary antibodies, including peroxidase AffiniPure goat anti‐mouse IgG (DY60203) and peroxidase AffiniPure goat anti‐rabbit IgG (DY60202), were obtained from DIYIBio.

### 4.11 Ex Vivo Fluorescence Imaging of Pulmonary Tissue for Analysing mCherry‐Labelled *S. aureus* Distribution

mCherry‐labelled *S. aureus* (1 × 10^8^ CFU in 20 µL PBS) were introduced intratracheally into isoflurane‐anesthetized C57BL/6 mice. At 1 and 10 d.p.i., mice were euthanized by cervical dislocation. Excised lungs were immediately imaged using an IVIS Spectrum system (PerkinElmer), with fluorescence acquisition at 570 nm excitation and 620 nm emission, all images were taken with a 5 s exposure time.

### 4.12 Isolation of Cell Membrane Proteins From Cell Cultures

Cell membrane proteins were isolated using the Mem‐PER Plus Membrane Protein Extraction Kit (Thermo Fisher Scientific, 89842) according to the manufacturer's instructions. The extracted protein fractions were divided into cryovials and stored at ‐80°C for subsequent analysis.

### 4.13 Electron Microscope

EVs and *EVs‐S. aureus* complexes were placed on 200‐mesh carbon‐coated copper grids. After a 5‐min incubation, excess suspension was wicked away with filter paper. Grids were negatively stained with 2% phosphotungstic acid (pH 6.8) for 30 s, then rinsed 10 s with ultrapure water. They were air‐dried in a laminar flow hood before imaging via TECNAI Spirit transmission electron microscope (FEI Company).

### 4.14 Synthesis of FNBP Domain‐Derived Peptides (D1‐D4)

The D1‐D4 peptides derived from the FNBP domain (32 kDa), expressed recombinantly in *E. coli* BL21(DE3) using the *S. aureus* FnBP sequence, were synthesized by Wuhan GeneCreate Biological Engineering Co., Ltd. For cell treatment, these peptides were used at 10 µg/mL for 30 min.

### 4.15 Quantitative Assessment of Total Adhesion and Intracellular *S. aureus*

After 3 h of *S. aureus* infection, the supernatant was removed, and cells were washed twice with PBS. Cells were then lysed with 1 mL of ice‐cold hypotonic buffer (0.1% Triton X‐100 in water) to release intracellular bacteria, which were quantified by the dilution plating method.

### 4.16 Quantitative Assessment of Intracellular *S. aureus*

After 3 h of *S. aureus* infection, the supernatant was removed, and cells were washed twice with PBS. Fresh complete medium with 20 µg/mL lysostaphin (Sigma, L7386) was added, and cells were incubated at 37°C, 5% CO_2_ for 30 min. The supernatant was then removed, and cells were rinsed with pre‐warmed (37°C) PBS without antibiotics. Cells were lysed with 1 mL of ice‐cold hypotonic buffer (0.1% Triton X‐100 in water), and intracellular bacteria were released and counted by dilution plating.

### 4.17 Transfection

To achieve targeted gene knockdown, lentiviral and adeno‐associated virus (AAV) systems were employed for in vitro and in vivo studies, respectively. For the in vitro studies, A549 cells were transduced with lentiviral vectors expressing shRNAs against Fibronectin (sh‐FN; Shanghai JiKai Gene) or Rab27a (sh‐Rab27a; Shanghai Hanbio Technology). A non‐targeting shRNA (sh‐NC) was used as a control. All transductions were carried out at a multiplicity of infection (MOI) of 10 with 4 µg/mL polybrene. Successfully transduced cells were selected with 1 µg/mL puromycin and subsequently isolated as monoclonal populations. For the in vivo knockdown, mice received an intratracheal injection of AAV6 (2.5 × 10^11^ vector genomes) carrying an shRNA targeting Rab27a under the control of the SP‐C promoter 4 weeks prior to *S. aureus* infection to deplete Rab27a specifically in lung tissue.

### 4.18 ELISA Assay

A 10% (w/v) lung tissue homogenate was made in ice‐cold saline (0.9% NaCl) and centrifuged at 3000 × *g* for 10 min at 4°C. The supernatant was collected, and IL‐1β, IL‐6 and TNF‐α levels were measured using species‐specific ELISA kits (GEM0001 for IL‐1β, GEM0002 for IL‐6, GEM0004 for TNF‐α; Wuhan Servicebio Technology Co., Ltd., China) per the manufacturer's instructions.

### 4.19 RNA Extraction and Quantitative Real‐Time PCR

Total RNA was extracted from tissue samples using TRIzol Reagent (Mmouse Biotechnology, MI00617) and quantified via a NanoDrop 2000 spectrophotometer (Thermo Fisher Scientific). Reverse transcription was conducted in 20 µL reactions containing 10 µg of total RNA using the SweScript All‐in‐One First‐Strand cDNA Synthesis SuperMix (Servicebio, G3337). mRNA levels were quantified with SYBR Green Master Mix (Servicebio, G3326) on a CFX 96Touch system (Bio‐Rad). Relative gene expression was determined using the −2^ΔΔCt^ method, normalized to GAPDH. The primers used were: *S. aureus*‐16sRNA (Forward: 5’‐GTGGAGGGTCATTGGAAACTG‐3’; Reverse: 5’‐CGTTTACGGCGTGGACTACC‐3’) and GAPDH (Forward: 5’‐CCTCGTCCCGTAGACAAAATG‐3’; Reverse: 5’‐TGAGGTCAATGAAGGGGTCGT‐3’).

### 4.20 Flow Cytometry

For flow cytometry, cells from both BALF and lung single‑cell suspensions were analysed. BALF was obtained by instilling 1 mL of PBS into the trachea, filtered through a 40‑µm nylon strainer, and centrifuged (250 × *g*, 6 min, 4°C). The pellet was resuspended in 100 µL of PBS containing 2% FBS and stained with FITC‑conjugated anti‑mouse CD3 (Thermo Fisher Scientific, 11‑0032‑82, 1:100) and PE‑conjugated anti‑mouse CD19 (Thermo Fisher Scientific, 12‑0193‑82, 1:100) for 30 min at 37°C in the dark.

Lung single‑cell suspensions were prepared by mincing lung tissue, digesting with collagenase IV (1 mg/mL in RPMI 1640, 37°C, 60 min with gentle agitation), and filtering through a 70‑µm cell strainer. After erythrocyte lysis and washing, cells were stained with PE‑conjugated anti‑mouse CD326 (EpCAM) monoclonal antibody (Thermo Fisher Scientific, 11‑5791‑82, 1:100) and APC‑conjugated anti‑mouse F4/80 antibody (BioLegend, 123115, 1:100) under the same conditions. Unbound antibodies were removed by two washes with PBS containing 2% FBS.

All samples were analysed on a CytoFLEX LX flow cytometer (Beckman Coulter), and data were processed using FlowJo software (v10.6.2). In selected experiments, EpCAM‑positive epithelial cells were isolated by sorting on a Sony MA900 cell sorter for downstream applications.

### 4.21 Nanoscale Flow Cytometry Analysis of EV Surface Markers

The surface protein marker of EVs was analysed using nanoscale flow cytometry (NanoFCM N30E). For analysis, 30 µL aliquots of PBS‐diluted EVs were stained in the dark at 37°C for 30 min with specific antibodies: FITC‑conjugated anti‐EpCAM antibody (Thermo Fisher Scientific, 11‐5791‐82, 1:100), FITC‑conjugated anti‐Ly6G antibody (BD Biosciences, 551460, 1:100), or FITC‑conjugated anti‐F4/80 antibody (Elabscience, E‐AB‐F0995C, 1:20), Unbound antibodies were removed by two rounds of ultracentrifugation (150,000 × *g*, 4°C), and the final pellets were resuspended in 50 µL of cold PBS. Before sample runs, the instrument was calibrated with standard particles, and EV samples were appropriately diluted to ensure stable fluidics. All data were acquired following standard protocols on the NanoFCM system.

### 4.22 Data Presentation and Statistical Analysis

Quantitative data are presented as mean ± SD. IBM SPSS Statistics 26 and GraphPad Prism 8.3.0 were used for data statistics and analysis. Survival curves were compared by log‐rank testing. Normality (Shapiro–Wilk) and variance homogeneity (Brown‐Forsythe) were verified. Parametric data with equal variances underwent: Unpaired two‐tailed *t*‐tests (pairwise comparisons); One‐way ANOVA with Tukey's post hoc test (multi‐group comparisons). Nonparametric data were analysed using: Mann‐Whitney U tests (pairwise); Kruskal–Wallis tests (multi‐group). Statistical significance was defined as *P* < 0.05. Detailed experimental replicates and specific statistical methods are annotated in figure legends. Sample sizes were based on established precedents (Dou et al. 2020; Kim et al. 2014; Kuang et al. 2023; Liu et al. 2020). All data points were included without exclusion.

## Author Contributions

Feng Ding, Shengkai Gong, Haotian Luo, Pei Wang, Geng Dou and Shiyu Liu conceived the project and designed the experiments. Feng Ding, Shengkai Gong, Dandan Wu, Lili Bao and Dingmei Zhang performed bacterial plate counts, immunofluorescence/H&E staining, and EV concentration assays. Haotian Luo and Geng Dou conducted the experiments, analysed the results and wrote the manuscript. Xiaoshan Yang and Zihan Li contributed to EV collection. Peijie He and Jiani Liu supervised animal studies. Yang Zhou and Zhengyan Wang performed histopathological analyses. Siying Liu and Pei Wang provided technical support. All authors read and approved the final manuscript.

## Conflicts of Interest

The authors declare no conflicts of interest.

## Supporting information



Supporting Information: jev270238‐sup‐0002‐Figures.pdf

Supporting Information: jev270238‐sup‐0001‐SuppMat.pdf

## Data Availability

The data that support the findings of this study are available from the corresponding author upon reasonable request.
