# Supplementary material for: Extracellular Vesicles Coordinate Bacterial Cloaking in Lung Epithelial Cells to Alleviate Acute Inflammatory Injury
Source: J Extracell Vesicles. 2026 Feb 8;15(2):e70238. doi: 10.1002/jev2.70238 (PMC12884003; doi:10.1002/jev2.70238)
Supplement: Supplementary file 1 — Supporting Information: jev270238‐sup‐0002‐Figures.pdf [file JEV2-15-e70238-s001.pdf]

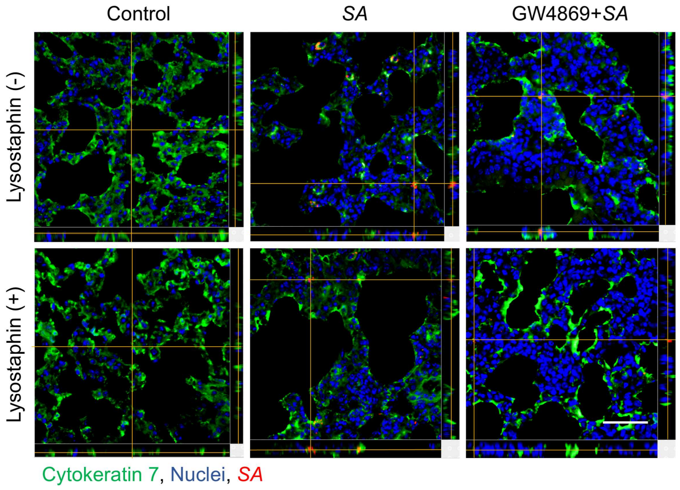

Supplementary Fig. 1 Lysostaphin eliminates luminal free and loosely attached bacteria.
Representative Z-stack fluorescence images showing mCherry-labeled *S. aureus* (red) in lung tissues across different groups. Scale bar, 50 μm.

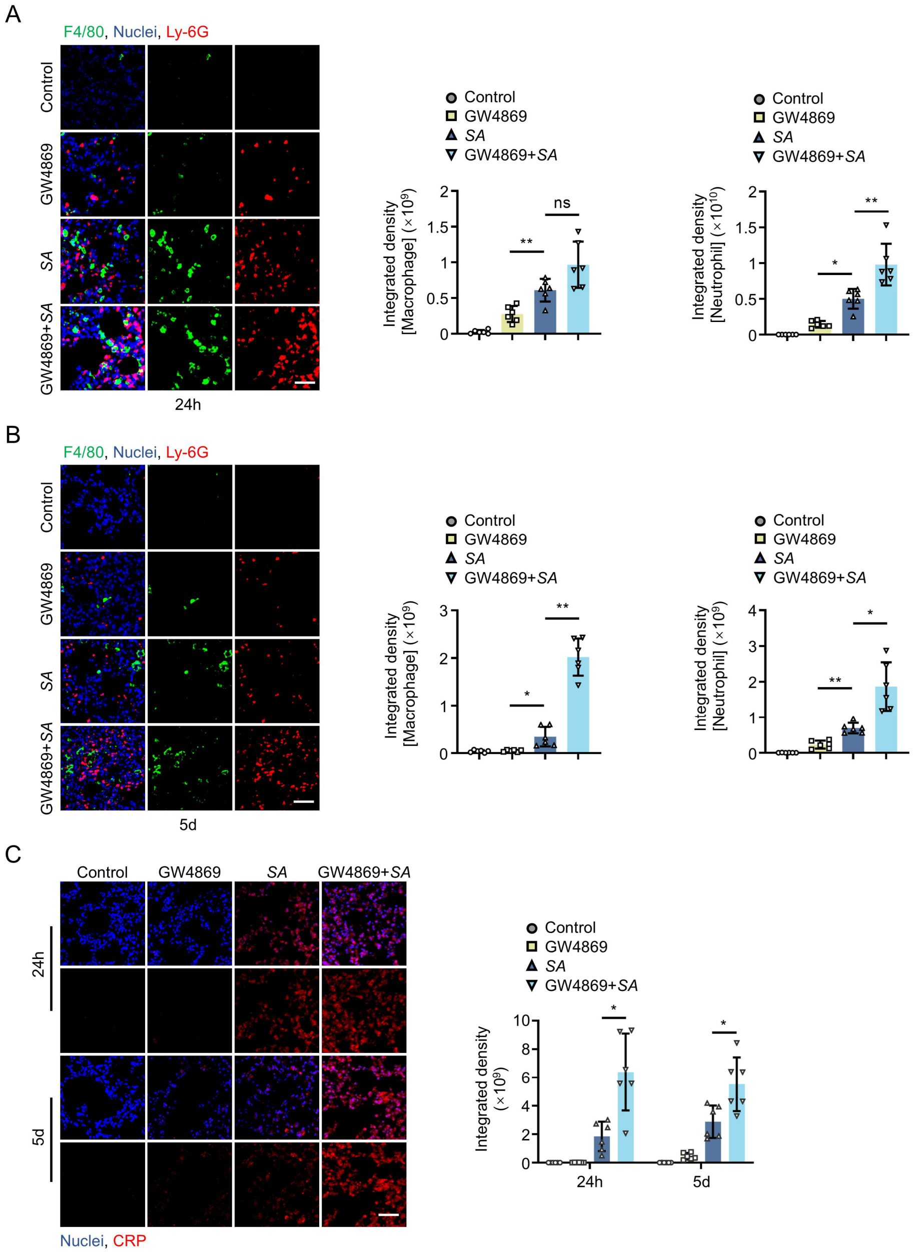

Supplementary Fig. 2 Inhibiting LuEV release intensifies pulmonary inflammation in *S. aureus*-induced pneumonia model.

A, B) Fluorescence images and quantitative analysis of F4/80$^+$ macrophage (green) and Ly-6G$^+$ neutrophil (red) distribution in mouse lung tissue at 24 h **(A)** and 5 d **(B)** post-*S. aureus* infection (n = 6). Scale bar, 50 µm. **C)** Fluorescence images and quantitative analysis of CRP expression (red) in mouse lung tissue at 24 h and 5 d post-*S. aureus* infection (n = 6). Scale bar, 50 µm. Data are presented as mean ± SD. *SA* denotes *Staphylococcus aureus*. Data were analyzed using one-way ANOVA with Tukey's post hoc test. *$p < 0.05$, **$p < 0.01$.

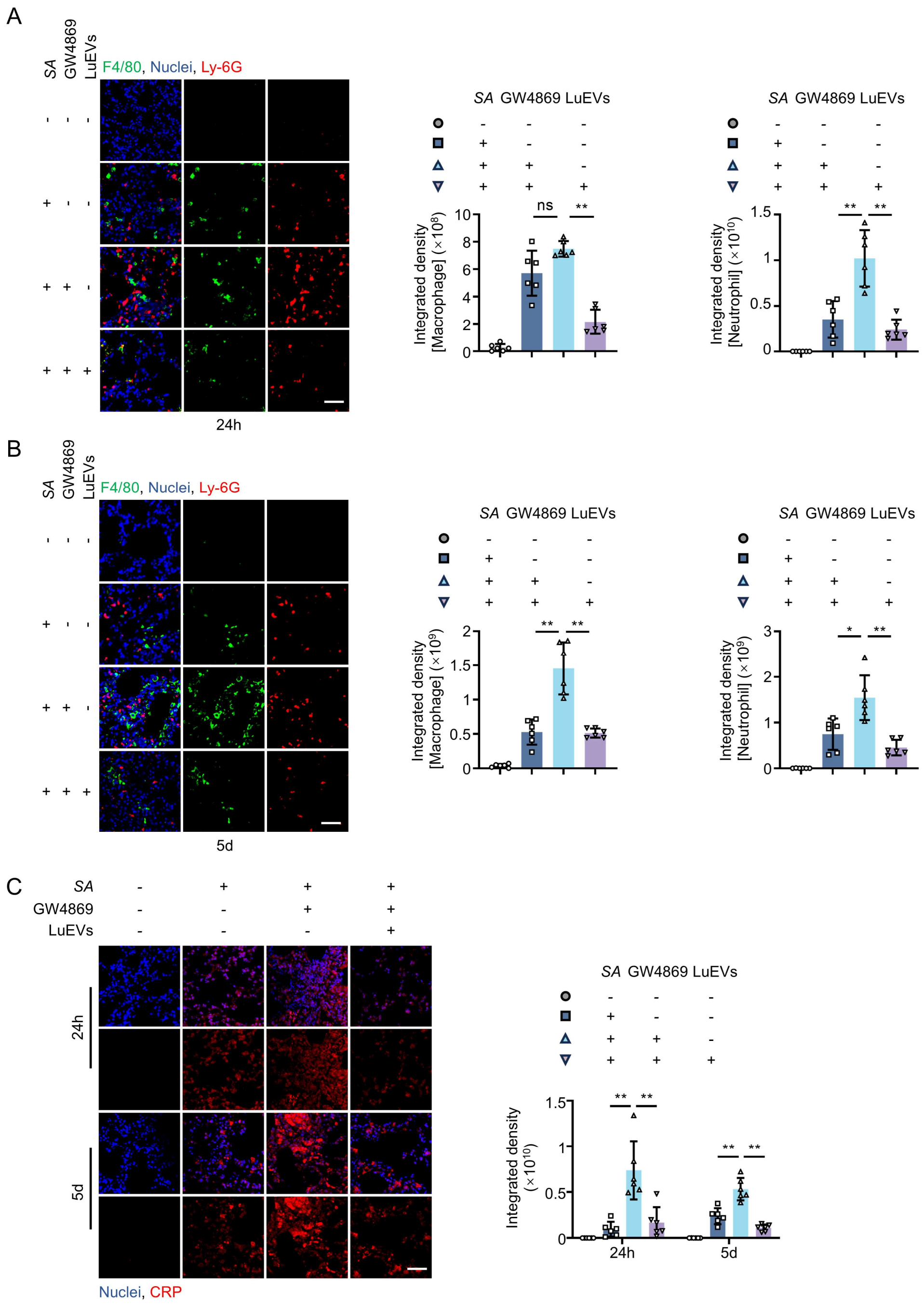

Supplementary Fig. 3 Re-supplementation of lung tissue-derived EVs (LuEVs) alleviates EV inhibition-induced pulmonary inflammation.

A, B) Fluorescence images and quantitative analysis of F4/80$^+$ macrophage (green) and Ly-6G$^+$ neutrophil (red) distribution in mouse lung tissue at 24 h **(A)** and 5 d **(B)** post-*S. aureus* infection (n = 6). Scale bar, 50 µm. **C)** Fluorescence images and quantitative analysis of CRP expression (red) in mouse lung tissue at 24 h and 5 d post-*S. aureus* infection (n = 6). Scale bar, 50 µm. *SA* denotes *Staphylococcus aureus*. LuEVs denote lung tissue cell-derived extracellular vesicles. Data are presented as mean ± SD. Data were analyzed using one-way ANOVA with Tukey's post hoc test. *$p < 0.05$, **$p < 0.01$.

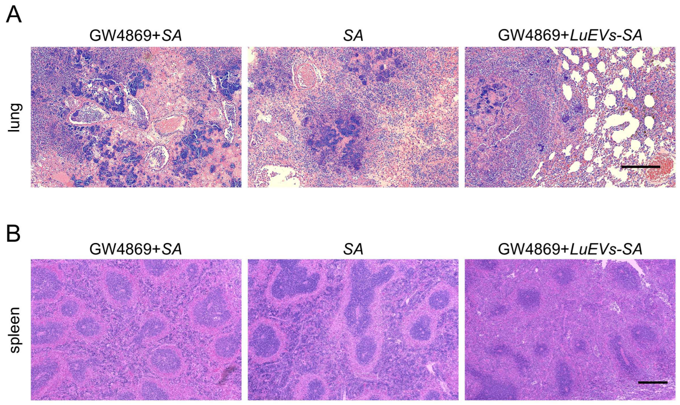

Supplementary Fig. 4 Analysis of mortality causes in *S. aureus*-induced pneumonia mouse model.

A, B) H&E staining of lungs **(A)** and spleens **(B)** from deceased mice in different groups. Scale bar, 200 μm **(A)**, 300 μm **(B)**. *SA* denotes *Staphylococcus aureus*. LuEVs denote lung tissue cell-derived extracellular vesicles.

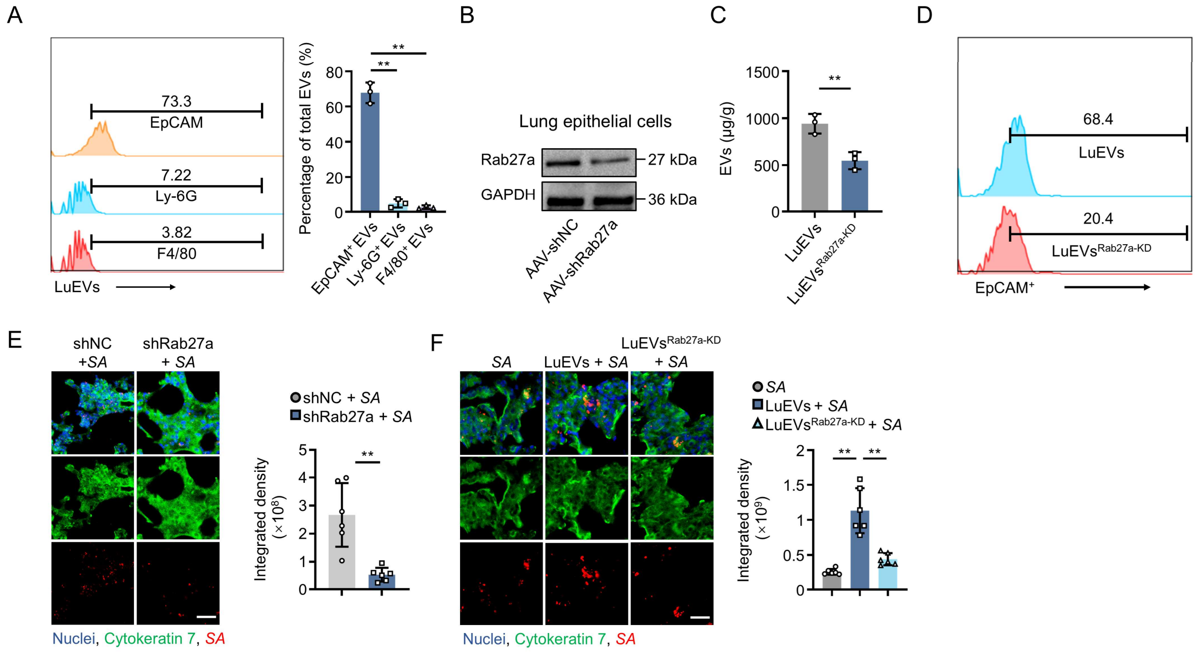

Supplementary Fig. 5 Epithelial derived-EVs constitute the major functional population of LuEVs during *S. aureus* infection.

A) Quantification of cell-specific surface markers on LuEVs in *S. aureus*-infected mice by nanoscale flow cytometry (n = 3). **B)** Validation of Rab27a knockdown in lung epithelial cells by Western blot (n = 3). **C)** Quantification of LuEV levels in infected mice after LECs-specific Rab27a konckdown (n = 3). **D)** Quantification of EpCAM^{+} population level in LuEVs after LECs-specific Rab27a konckdown by nanoscale flow cytometry (n = 3). **E)** Representative fluorescence images and quantitative analysis of intracellular *S. aureus* (red) level in infected lung tissue after LECs-specific Rab27a konckdown (n = 6). Scale bar, 25 μm. **F)** Representative fluorescence images and quantitative analysis of *S. aureus* (red) level in cytokeratin 7^{+} LECs (green) in lung tissue of infected mice from different groups at 24 h.p.i. (n = 6). Scale bar, 25 μm. AAV denotes Adeno-Associated Virus. *SA* denotes *Staphylococcus aureus*. LuEVs denotes lung tissue-derived extracellular vesicles. LuEVs$^{\text{Rab27a-KD}}$ denotes LuEVs isolated from the AAV-shRab27a mice. Data are presented as mean ± SD. Statistical significance was assessed by one-way ANOVA with Tukey's post hoc test. *$p < 0.05$, **$p < 0.01$.

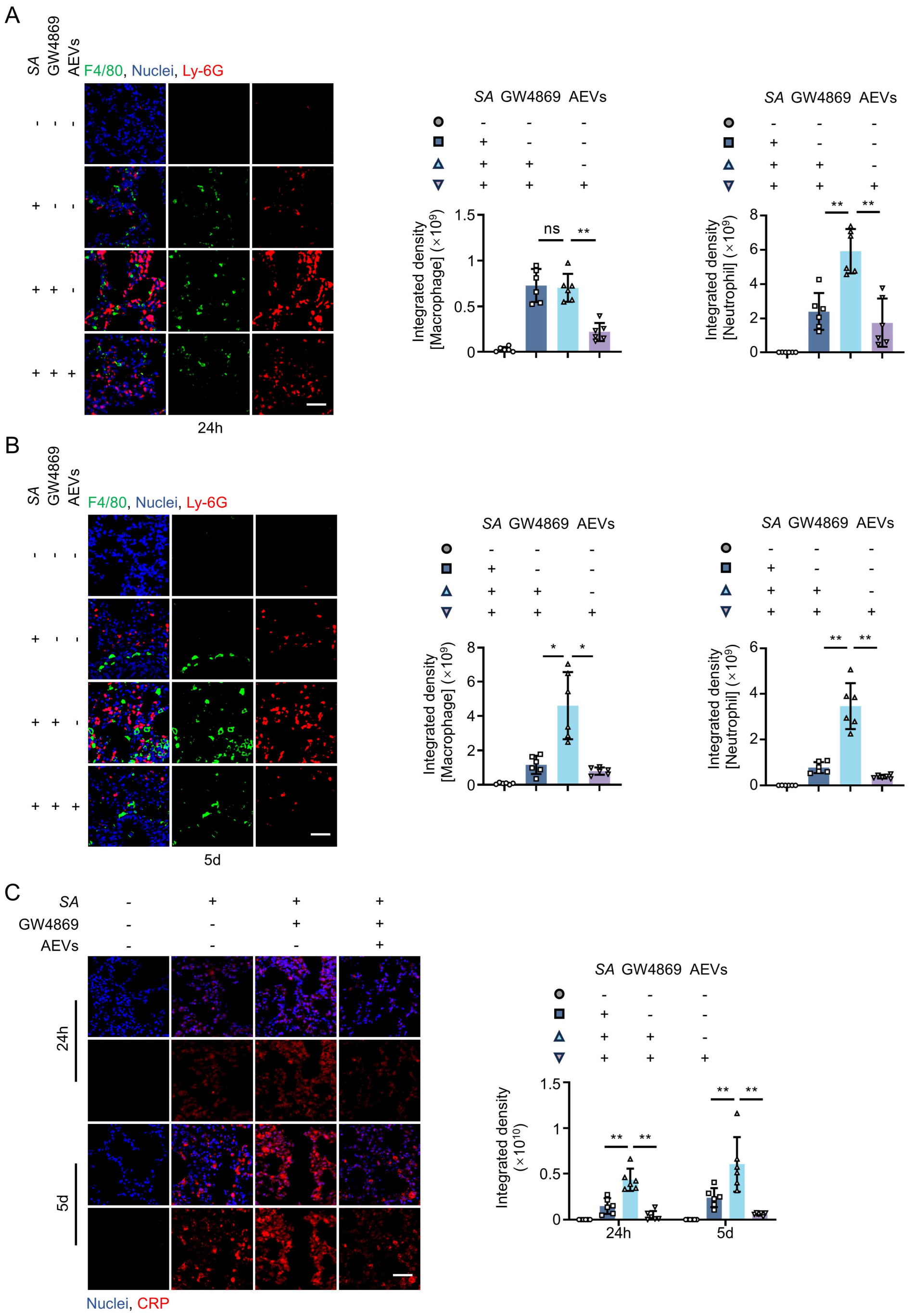

Supplementary Fig. 6 Re-supplementation of AEVs alleviates EV inhibition-induced pulmonary inflammation.

A, B) Fluorescence images and quantitative analysis of F4/80$^+$ macrophage (green) and Ly-6G$^+$ neutrophil (red) distribution in mouse lung tissue at 24 h **(A)** and 5 d **(B)** post-*S. aureus* infection (n = 6). Scale bar, 50 µm. **C)** Fluorescence images and quantitative analysis of CRP expression (red) in mouse lung tissue at 24 h and 5 d post-*S. aureus* infection (n = 6). Scale bar, 50 µm. *SA* denotes *Staphylococcus aureus*. AEVs denote A549 cell-derived extracellular vesicles. Data are presented as mean ± SD. Data were analyzed using one-way ANOVA with Tukey's post hoc test. $^*p < 0.05$, $^{**}p < 0.01$.

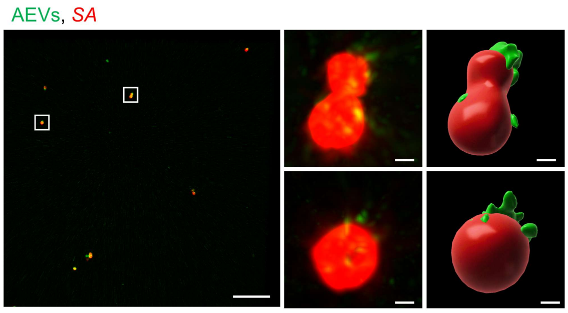

Supplementary Fig. 7 SR-SIM analysis of EV-bacteria interaction.

Representative SR-SIM micrographs and 3D reconstructions of mCherry-labeled *S. aureus* (red) incubated with PKH67-labeled EVs (green) from infected A549 cells (n = 3). Scale bars, 10 μm (low-magnification); 200 nm (high-magnification). AEVs denotes A549 cell-derived extracellular vesicles.

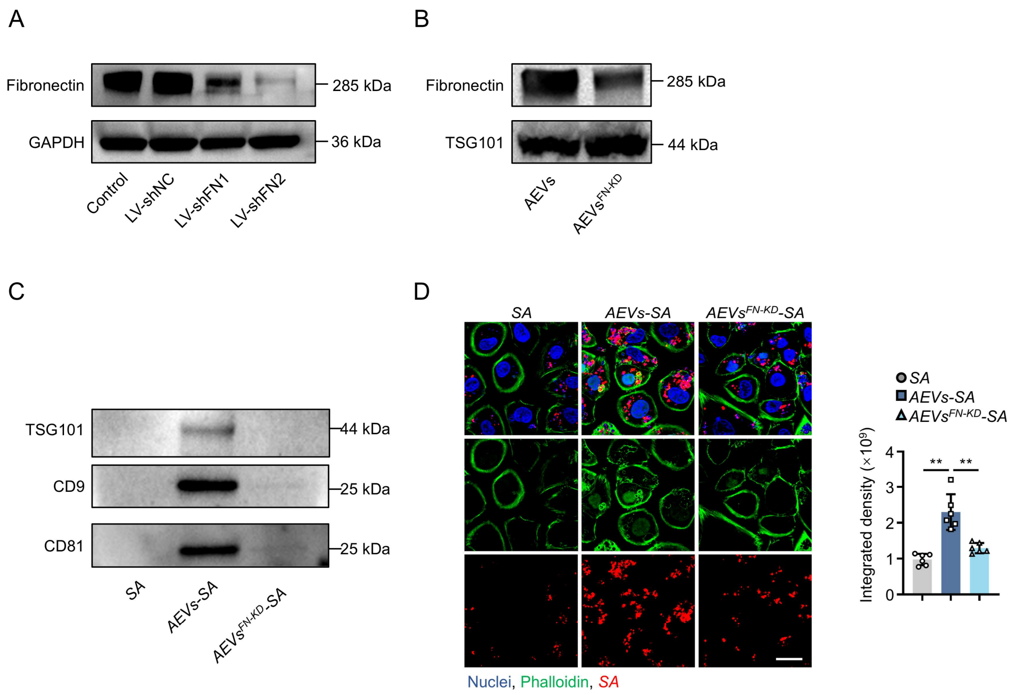

Supplementary Fig. 8 Fibronectin (FN) on AEVs mediates specific binding to bacteria.

A) Western blot analysis validating FN knockdown in A549 cells (n = 3). **B)** Western blot analysis of fibronectin level on AEVs$^{FN\text{-}KD}$ (n = 3). **C)** Western blot analysis of TSG101, CD9 and CD81 on *S. aureus* pre-incubated with different EVs (n = 3). **D)** Representative fluorescence images and quantitative analysis of intracellular *S. aureus* (red) level in A549 cells across different groups (n = 6). Scale bar, 25 µm. LV denotes lentivirus. *SA* denotes *Staphylococcus aureus*. AEVs denotes A549 cell-derived extracellular vesicles. AEVs$^{FN\text{-}KD}$ denotes EVs derived from FN-knockdown A549 cells. *AEVs-SA* denotes *AEVs-S. aureus* complexes. Data are presented as mean ± SD. Statistical significance was assessed by one-way ANOVA with Tukey's post hoc test. *$p < 0.05$, **$p < 0.01$.

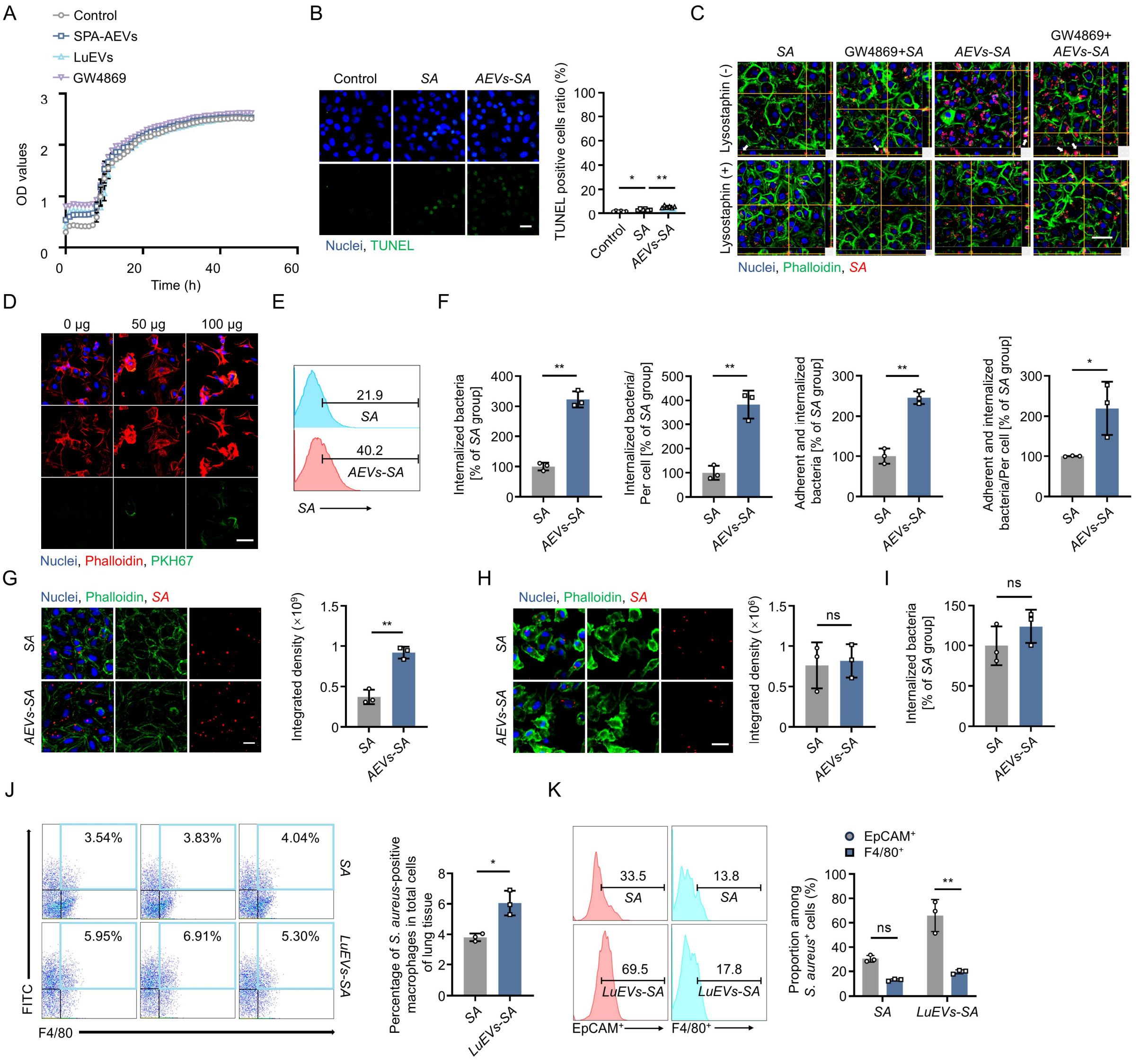

Supplementary Fig. 9 *AEV-SA* complexes increased epithelial invasion without affecting macrophage phagocytosis.

A) Proliferation rate of *S. aureus* treated with different EVs and GW4869 (n = 3), OD=600nm. **B)** Fluorescence images and quantitative analysis of TUNEL staining in *S. aureus*-infected A549 cells across different groups (n = 6). Scale bar, 25 µm. **C)** Representative Z-stack fluorescence images showing *S. aureus* (red) level in A549 cells across different groups. Scale bar, 50 µm. **D)** Fluorescence images of PKH67-labeled AEVs (green) in A549 cells (red) at various concentrations. Scale bar, 50 µm. **E)** Flow cytometry analysis of *S. aureus* level in A549 cells across different groups. **F)** Quantification of *S. aureus* level in A549 cells by plate counts, showing both the internalized bacteria (lysostaphin-treated) and the total cell-associated population (adherent and internalized, lysostaphin-untreated) (n = 3). **G)** Fluorescence images and quantitative analysis of total adherent and internalized *S. aureus* (red) level in A549 cells (green) treated by SPA-AEVs (n = 3). Scale bar, 25 µm. **H, I)** Fluorescence images and quantitative analysis **(H)** and bacterial plate counts **(I)** of phagocytosed bacteria in BMDMs (n = 3). Scale bar, 25 µm **(H)**. **J)** Flow cytometry analysis of *S. aureus*-positive macrophages in single-cell suspensions prepared from lung tissue. **K)** Flow cytometry analysis of the proportion of epithelial cells (EpCAM^{+}) and macrophages (F4/80^{+}) among all GFP-labeled *S. aureus* (FITC)$^{+}$ cells in lung tissue. *SA* denotes *Staphylococcus aureus*. *AEVs-SA* indicates *AEVs-*

S. aureus complexes. LuEVs denotes lung tissue-derived extracellular vesicles. Data are presented as mean ± SD. Statistical significance was assessed by one-way ANOVA with Tukey's post hoc test. *$p<0.05$, **$p<0.01$.

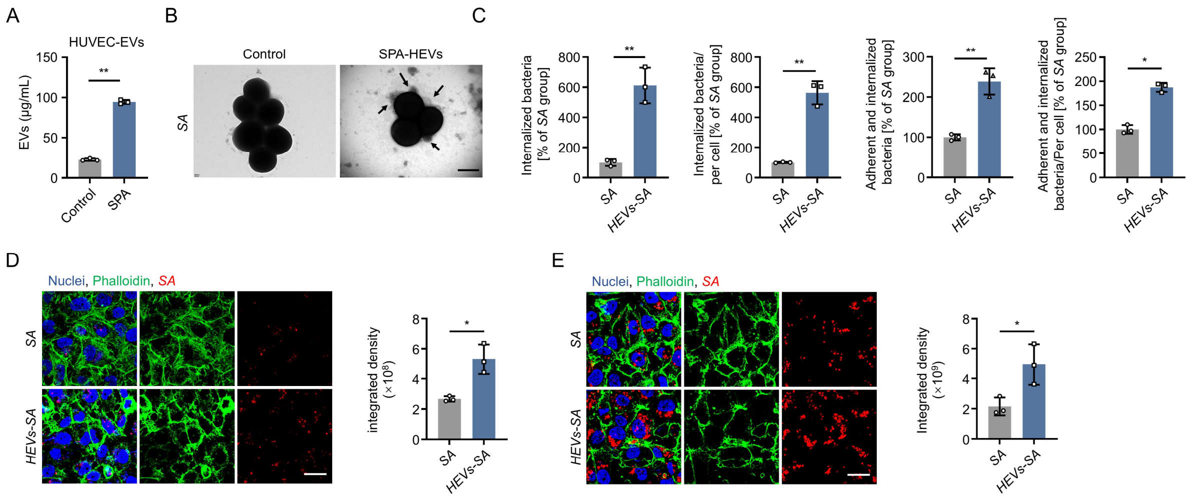

Supplementary Fig. 10 SPA-treated endothelial cell-derived EVs facilitate bacterial binding and internalization in HUVECs.

A) Quantification of EV levels in supernatant of SPA-stimulated HUVECs (n = 3). **B)** TEM images of *S. aureus* incubated with HEVs (n = 3). Scale bar, 500 nm. Black spherical structures are *S. aureus*. The black arrows indicate EVs bound to the bacterial surface. **C)** Quantification of *S. aureus* in HUVECs by plate counts, showing both the internalized bacteria (lysostaphin-treated) and the total cell-associated population (adherent and internalized, lysostaphin-untreated) (n = 3). **D, E)** Fluorescence images and quantitative analysis of internalized (lysostaphin-treated) **(D)** and total adherent and internalized (lysostaphin-untreated) **(E)** *S. aureus* (red) level in HUVECs post-infection (n = 3). Scale bar, 25 μm. *SA* denotes *Staphylococcus aureus*. HEVs denotes HUVEC cell-derived extracellular vesicles. *HEVs-SA* indicates *HEVs-S. aureus* complexes. Data are presented as mean ± SD. Statistical significance was assessed by one-way ANOVA with Tukey's post hoc test. *p<0.05, **p<0.01.

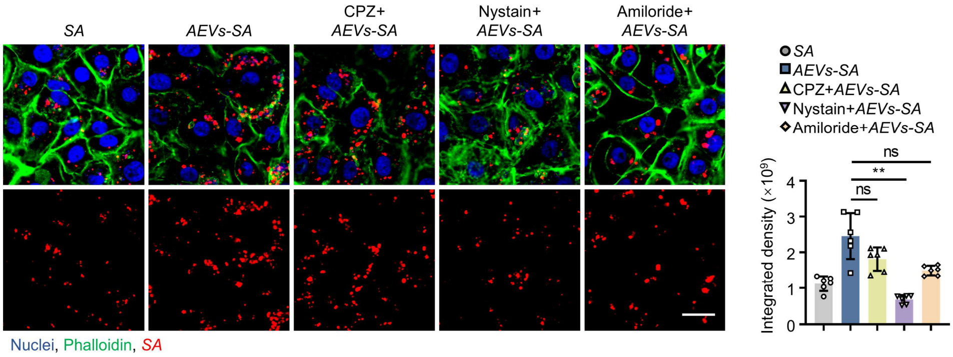

Supplementary Fig. 11 *AEVs-SA* complexes enter lung epithelial cells predominantly via the caveolin-dependent pathway.

Representative fluorescence images and quantitative analysis of intracellular *S. aureus* (red) level in A549 cells across different treatment groups (n = 6). Scale bar, 25 μm. AEVs denotes A549 cell-derived extracellular vesicles. *AEVs-SA* denotes *AEVs-S. aureus* complexes. Data are presented as mean ± SD. Statistical significance was assessed by one-way ANOVA with Tukey's post hoc test. *$p < 0.05$, **$p < 0.01$.
