# Supplementary material for: Extracellular Vesicles Coordinate Bacterial Cloaking in Lung Epithelial Cells to Alleviate Acute Inflammatory Injury
Source: J Extracell Vesicles. 2026 Feb 8;15(2):e70238. doi: 10.1002/jev2.70238 (PMC12884003; doi:10.1002/jev2.70238)
Supplement: Supplementary file 2 — Supporting Information: jev270238‐sup‐0001‐SuppMat.pdf [file JEV2-15-e70238-s002.pdf]

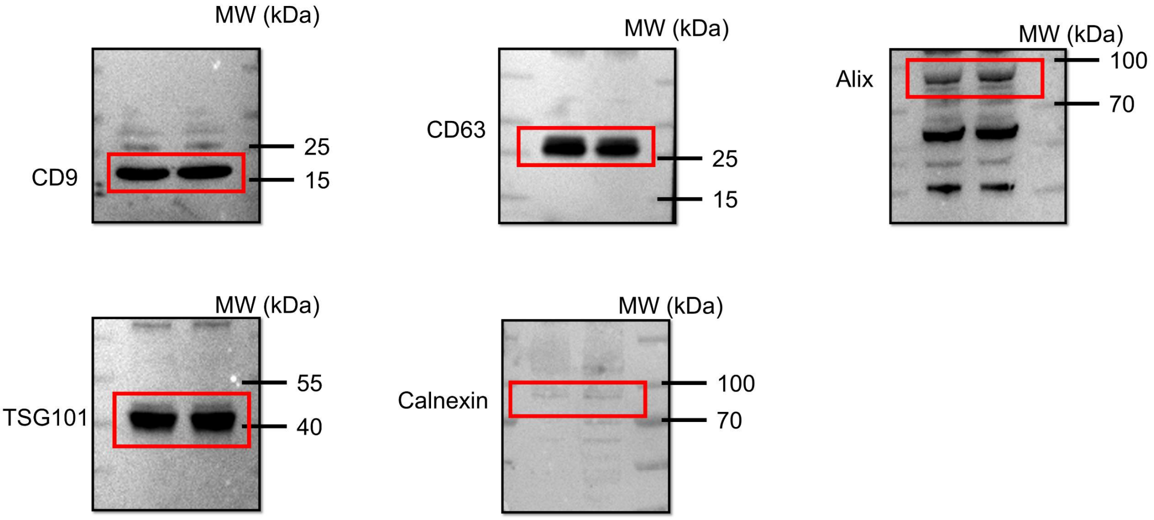

Western blot band in Fig. 2D

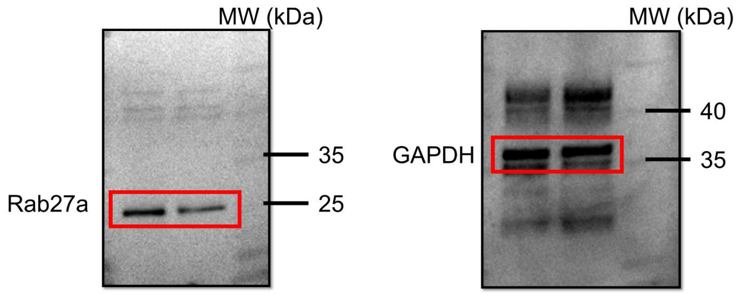

Western blot band in Supplementary Fig. 5B

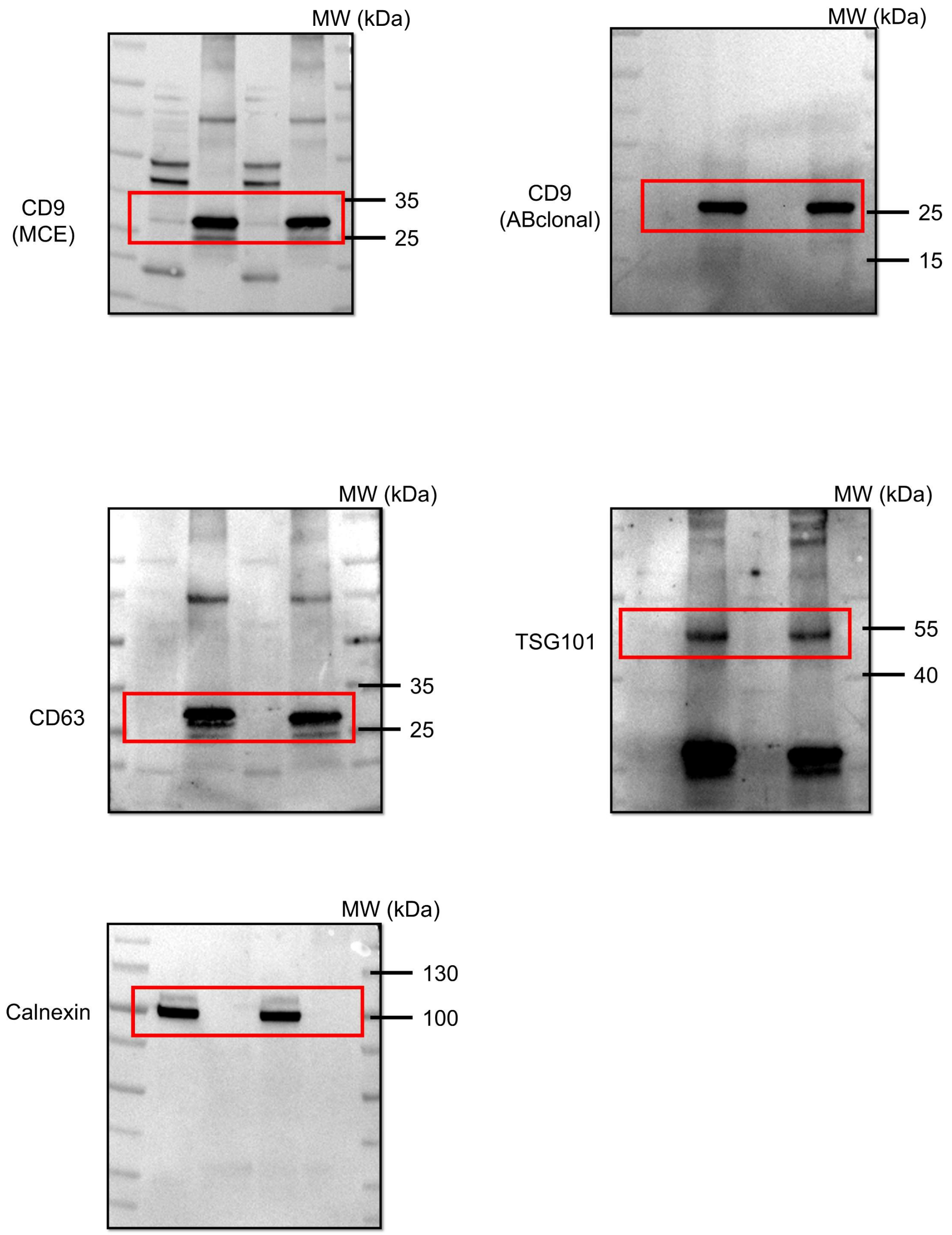

Western blot band in Fig. 3D

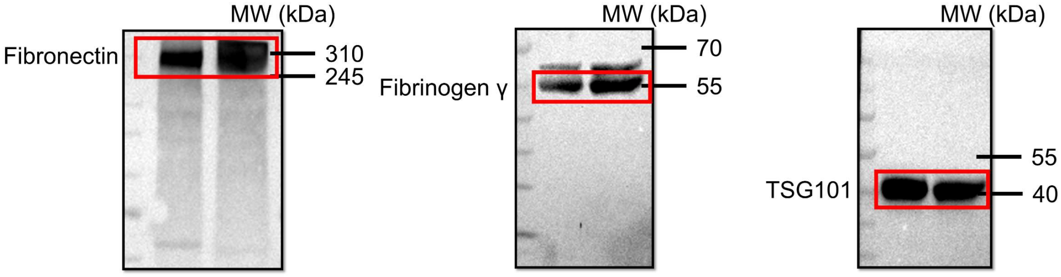

Western blot band in Fig. 4A

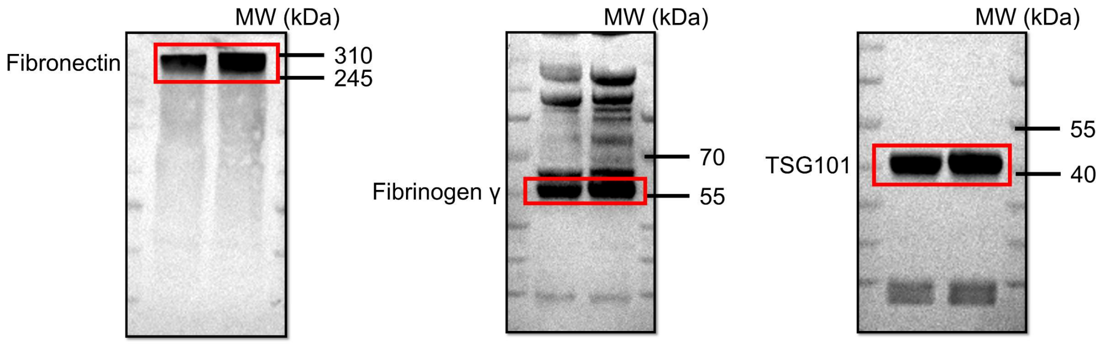

Western blot band in Fig. 4B

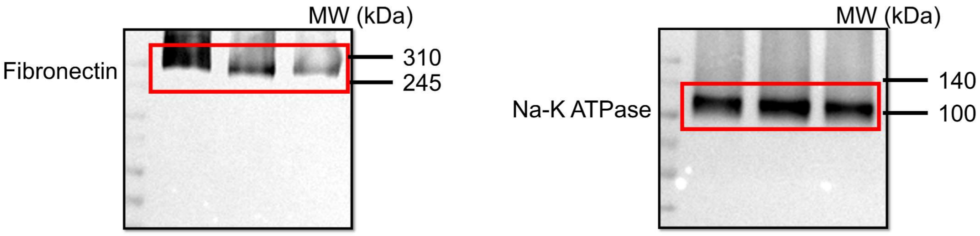

Western blot band in Fig. 4D

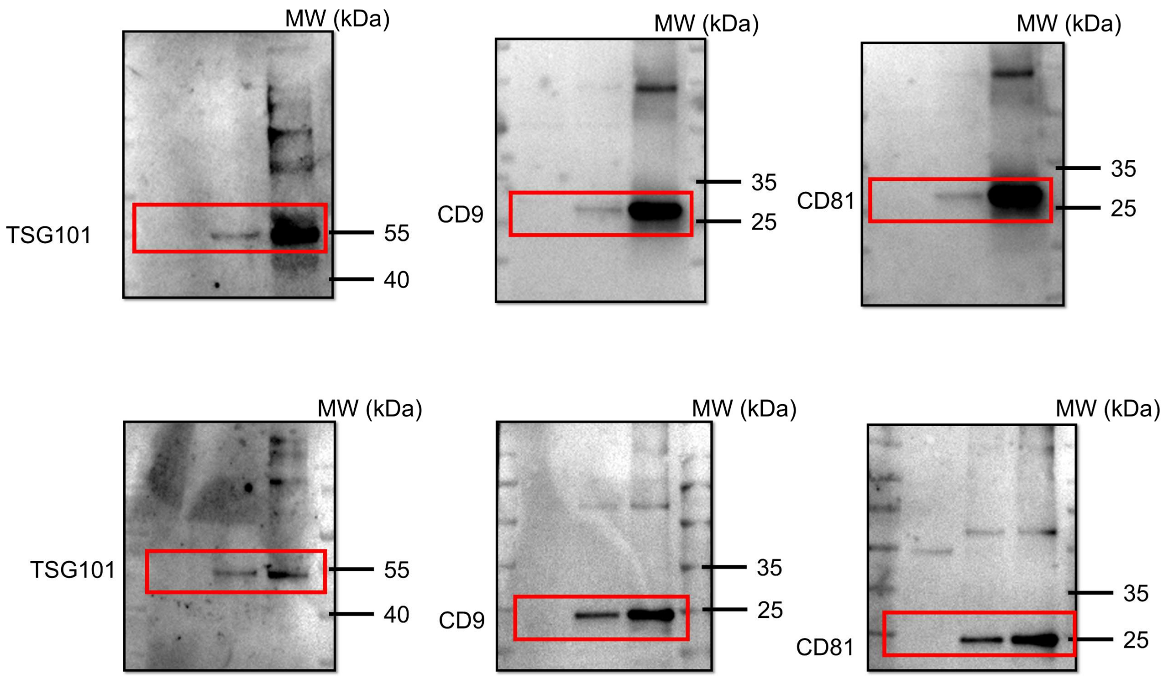

Western blot band in Fig. 4J

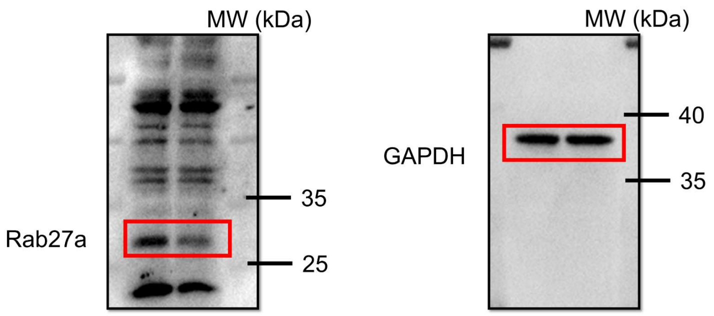

Western blot band in Fig. 4O

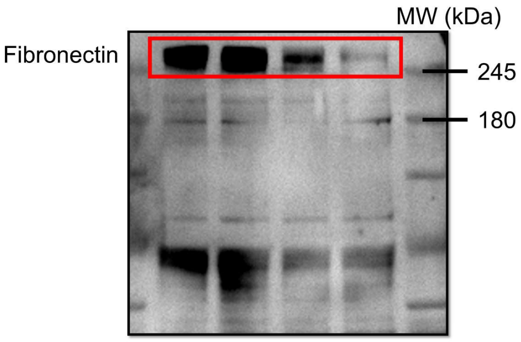

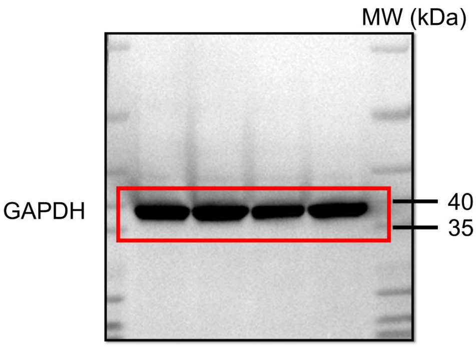

Western blot band in Supplementary Fig. 8A

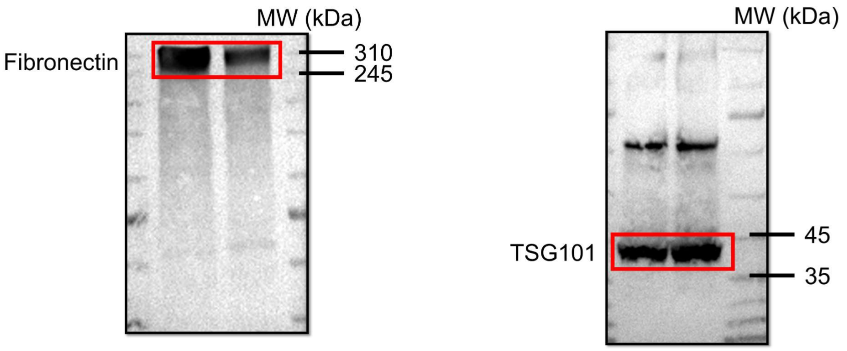

Western blot band in Supplementary Fig. 8B

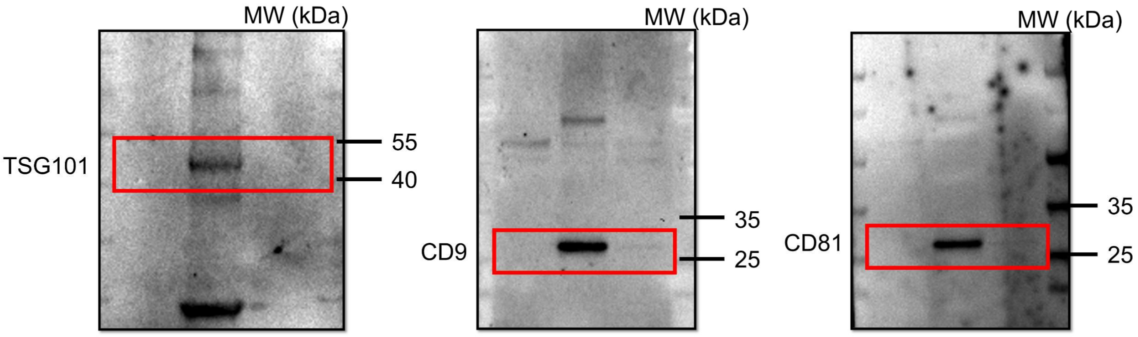

Western blot band in Supplementary Fig. 8C

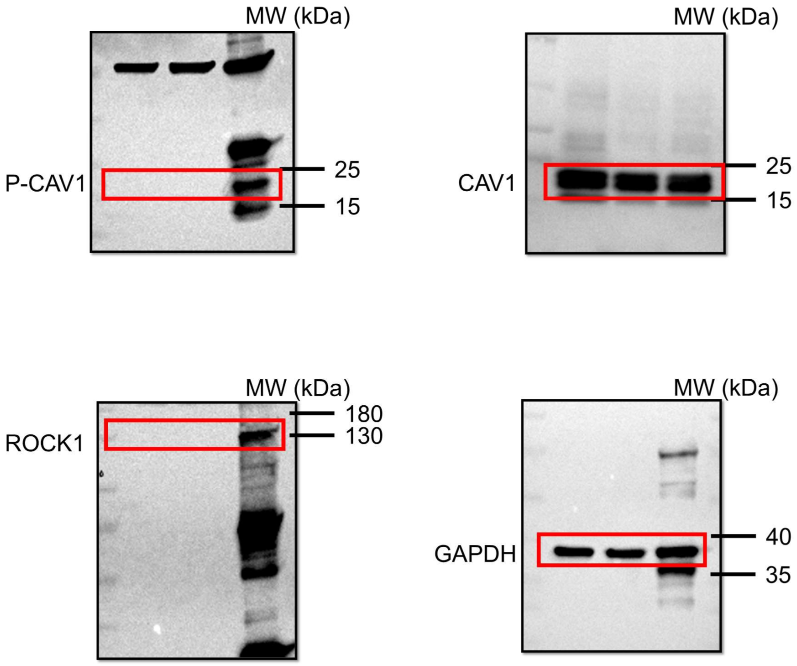

Western blot band in Fig. 5D

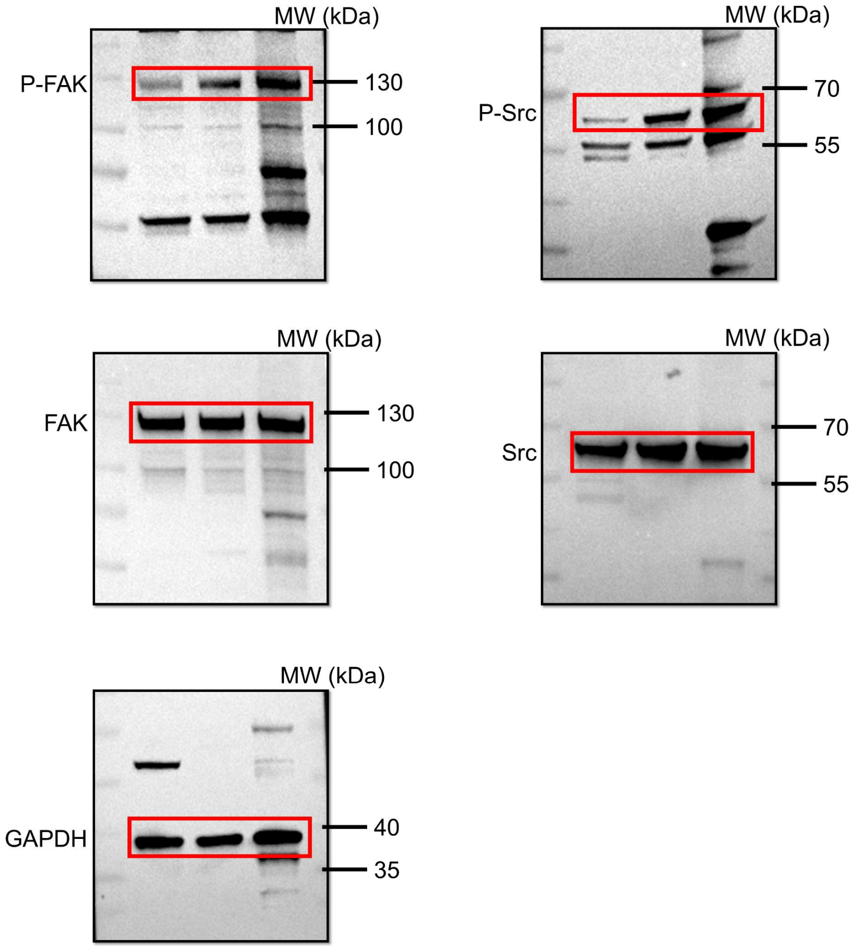

Western blot band in Fig. 5E

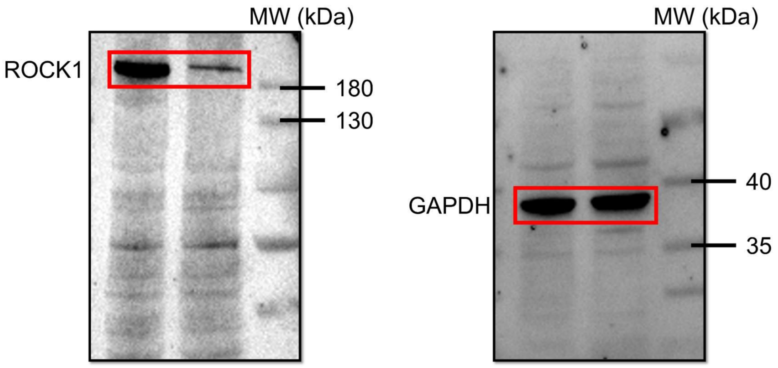

Western blot band in Fig. 5K
